# Laser dissection‐assisted phloem transcriptomics highlights the metabolic and physiological changes accompanying clubroot disease progression in oilseed rape

**DOI:** 10.1111/tpj.17156

**Published:** 2024-11-22

**Authors:** Sara Blicharz, Karolina Stefanowicz, William Truman, Aneta Basińska‐Barczak, Deeksha Singh, Anna Kasprzewska, Nuria de Diego, Ondřej Vrobel, Sanja Ćavar Zeljković, Petr Tarkowski, Robert Malinowski

**Affiliations:** ^1^ Institute of Plant Genetics Polish Academy of Sciences ul. Strzeszyńska 34 Poznań 60‐479 Poland; ^2^ Czech Advanced Technology and Research Institute (CATRIN) Palacký University Olomouc Olomouc Czech Republic

**Keywords:** clubroot, *Plasmodiophora brassicae*, phloem, laser dissection transcriptomics, oilseed rape, *Brassica napus*

## Abstract

*Plasmodiophora brassicae*, a soil‐borne biotroph, establishes galls as strong physiological sinks on Brassicaceae plants including *Brassica napus* and *Arabidopsis thaliana*. We compare transcriptional profiles of phloem dissected from leaf petioles and hypocotyls of healthy and infected *B. napus* plants. Our results highlight how pathogenesis accompanies phloem‐mediated defence responses whilst exerting a strong influence on carbon–nitrogen (C–N) economy. We observe transcriptional changes indicating decreased aliphatic glucosinolate biosynthesis, fluctuating jasmonic acid responses, altered amino acid (AA) and nitrate transport, carbohydrate metabolism and modified cytokinin responses. Changes observed in phloem‐dissected from upper versus lower plant organs point to phloem as a conduit in mediating C–N repartitioning, nutrition‐related signalling and cytokinin dynamics over long distances during clubroot disease. To assess changes in physiology, we measured AAs, sugars and cytokinins, in phloem exudates from *B. napus* plants. Despite the decrease in most AA and sucrose levels, isopentyl‐type cytokinins increased within infected plants. Furthermore, we employed Arabidopsis for visualising promoter activities of *B. napus* AA and N transporter orthologues and tested the impact of disrupted cytokinin transport during *P. brassicae*‐induced gall formation using *Atabcg14* mutants. Our physiological and microscopy studies show that the host developmental reaction to *P. brassicae* relies on cytokinin and is accompanied by intense nitrogen and carbon repartitioning. Overall, our work highlights the systemic aspects of host responses that should be taken into account when studying clubroot disease.

## Significance Statement

This work describes transcriptomic and physiological changes in phloem tissue observed in rapeseed (*Brassica napus*) plants infected with *Plasmodiophora brassicae*—the causal agent of clubroot disease. It has been shown that early stages of the disease are accompanied by changes in the phloem transcriptional profiles and chemical content, which suggests the redirection of compounds rich in C and N towards the pathogen sink, and the mediation of postinfectious organ growth responses by cytokinins.

## INTRODUCTION

*Plasmodiophora brassicae* (Woronin, 1878) is a biotrophic protist plant pathogen that establishes its physiological niche by triggering the formation of characteristic galls on the underground plant parts, hence the disease name—clubroot. Infection leads to dramatic developmental and physiological reprogramming in plants. The developing clubroot gall acts as a pathogen sink where the extensive delivery and subsequent consumption of host nutrients occurs (Malinowski et al., 2019). To facilitate this, the pathogen hijacks host developmental programs to stimulate the metabolic activity of cells by activation of proliferation and subsequently endoreduplication (Olszak et al., 2019). Previous work has shown that *P. brassicae* has the ability to increase the abundance of Sugars Will Eventually be Exported Transporters (SWEETs) at the sink to facilitate apoplastic active unloading of sugars in *Arabidopsis thaliana* and *Brassica rapa* (Kong et al., 2024; Li et al., 2018; Walerowski et al., 2018; Zhang et al., 2019). Impairment of this mechanism resulted in a delay in clubroot disease progression (Walerowski et al., 2018). *P. brassicae* also changes the anatomy of phloem cells, presumably increasing this way the rate of phloem transport (Gustafsson et al., 1986; Malinowski et al., 2012). Interestingly, it was observed that combined knock‐out mutations in *SWEET11* and *SWEET12* transporters did not have a detrimental impact on diseased plants whereas *brx‐2*, *cvp2‐1 cvl1‐1* and *ops‐2* mutants that could not respond to clubroot disease with enhanced phloem development died prematurely (Walerowski et al., 2018). This raises the question about processes other than simple carbohydrate repartitioning and unloading at the pathogen site, which may be mediated over long distances by phloem during clubroot disease. Based on the current state of knowledge regarding transport of metabolites, phytohormones, transcription factors and small RNA molecules (Turgeon & Wolf, 2009) we can assume, that long‐distance coordination has a tremendous impact on the whole plant–microorganism interaction. *P. brassicae* almost exclusively infects the root system and hypocotyl of the plant, therefore wholescale reprogramming of host nutrient distribution must be mediated over long distances (Malinowski et al., 2019). To date, little attention has been paid to the coordination of the host responses between the upper and lower parts of the plant. Such coordination is crucial for the host, but the pathogen also needs to induce significant adjustments in the physiology and metabolism within the aerial part of the infected plant. To investigate the changes occurring across long‐distance transport in clubroot‐infected plants, we decided to perform transcriptomic profiling of phloem cells collected from two distinct regions of the host: petioles of fully expanded leaves representing the source and hypocotyls representing the sink, which is also the site of gall formation in infected plants. Due to the fact that oilseed rape (OSR) has the highest economical value amongst *P. brassicae* hosts, we choose this as the primary model for our studies. This work was performed using laser capture microdissection microscopy (LCM) of phloem cell populations from mock‐ and *P. brassicae*‐inoculated *B. napus* plants followed by paired‐end RNA‐sequencing. This allowed us to gain a deeper insight into the subtle transcriptional changes that occur in this tissue which may typically be masked by the higher population of other cell types when whole‐organ transcriptomic analysis is performed. Inspection of patterns observed at both, 7‐ and 12‐days post‐inoculation (DPI), enabled us to view the dynamic changes occurring within the phloem content which revealed both, defence reactions typical of biotrophic infections, as well as coordinated changes facilitating carbon and nitrogen repartitioning. To delve further into the subject of pathogen‐driven nutrient allocation, we analysed cytokinin, amino acid (AA) and sugar content in phloem sap collected from mock‐ and *P. brassicae*‐inoculated *B. napus* plants. We found that despite a decline in the rate of photosynthesis and the concomitant decrease in the ability to synthesise nutrients which further limits the host's capacity to support their endogenous organs, *P. brassicae*‐induced developing galls act as the main consumption site and this physiological niche dominates the plant. Additionally, we then visualised induction of *B. napus* nitrate (*BnNPF7.2*) and amino‐acid transporter (*BnAAP1*) genes in Arabidopsis leaf petioles upon *P. brassicae* infection. Finally, for functionally dissecting the role of cytokinin transport in clubroot‐infected plants, we used *A. thaliana* cytokinin transporter mutant *abcg14* and tracked disease progression. Overall, our results describe the transcriptional changes at the level of phloem tissue observed during the early steps of clubroot disease progression reflective of systemic host reactions mediated over long distances within infected plants. Furthermore, from our results we can infer how phloem experiences both developmental and physiological reprogramming which is then manifested in the form of dynamic transcriptional and metabolic changes, supporting its pivotal role in modulating a constant dialogue between aerial and underground tissues of the host during clubroot disease.

## RESULTS

### Comparative transcriptomics, targeting phloem tissue, reveals distinct responses between colonised hypocotyls and non‐colonised upper parts of *P. brassicae* infected *B. napus*

**Figure 1:**
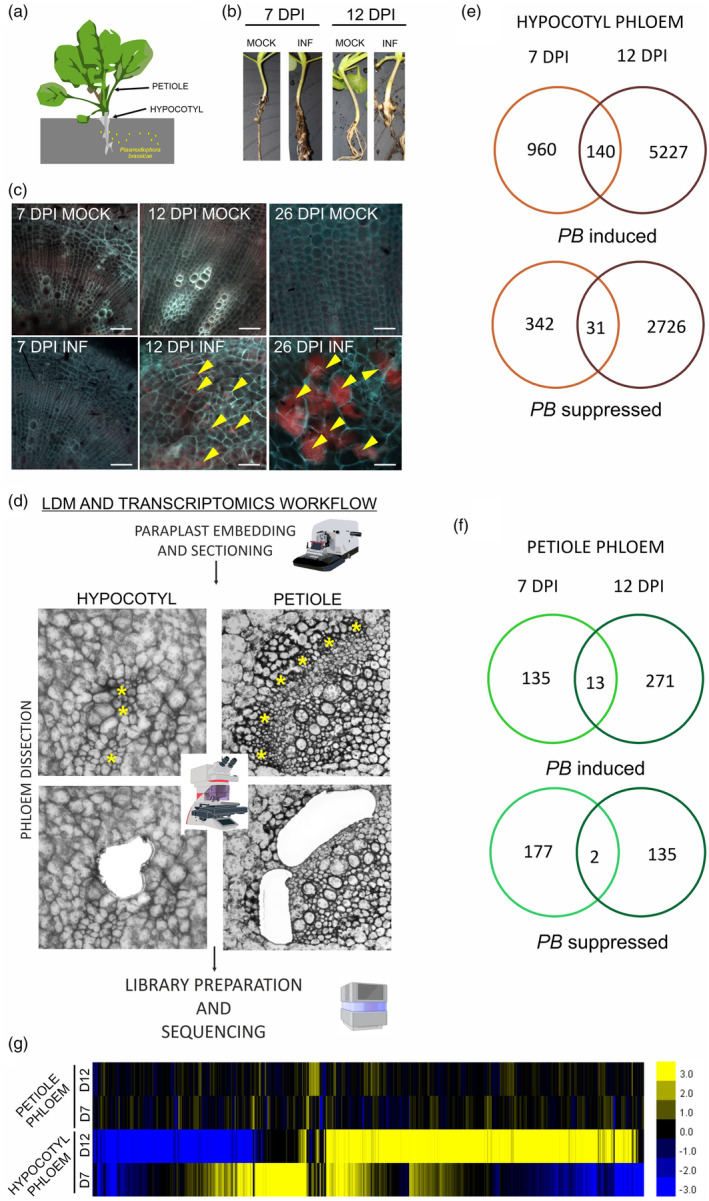
Phloem tissue transcriptional changes observed in *Plasmodiophora brassicae*‐inoculated *Brassica napus* reflect clubroot disease progression and diverse roles of the aboveground and belowground parts of the host plant in response to infection. (a) Scheme presenting the aboveground (petiole) and belowground (hypocotyl) material collected for phloem tissue isolation from *P. brassicae*‐infected plants. (b) *B. napus* mock‐treated (MOCK) and *P. brassicae*‐infected (INF) plants 7 and 12 DPI. (c) Hypocotyl cross sections of *B. napus* plants infected with *P. brassicae* 7 and 12 DPI stained with Nile Red. Scale bar represents 100 μm. Yellow arrowheads indicate cells colonised by the pathogen (red staining). (d) Workflow for laser‐capture microdissection (LDM) and transcriptomic analysis of phloem tissue. (e, f) Venn diagrams presenting the number of differentially expressed genes (DEGs) in the *P. brassicae‐*infected (e) hypocotyls and (f) petioles compared to the mock‐treated tissue 7 and 12 DPI. DEGs were split into PB‐induced and PB‐supressed genes. DEGs fold change ≥2, FDR ≤0.05. (g) Heatmap of log_2_ fold change values of the identified DEGs in the hypocotyl and petiole phloem samples from the *P. brassicae‐*infected *B. napus* plants compared to the mock‐treated plants 7 and 12 DPI. Upregulated genes are shaded in yellow and downregulated genes in blue. Significant DEGs were identified based on the thresholds of log_2_ ratio ≤−1 or ≥1 and a false discovery rate ≤0.05.

To distinguish between early responses, potentially involved in the detection of pathogen invasion, and later steps overlapping with cellular and developmental reprogramming leading to gall formation and susceptible *B. napus* host responses, we choose 7 and 12 DPI as the time points to sample (Figure 1a,b). Bi et al. (2016) demonstrated that owing to the ability of Nile Red to stain lipid droplets produced by the pathogen the presence of all developmental stages of *P. brassicae* can be visualised in host cells throughout the progression of clubroot disease. Staining revealed that at 12 DPI the majority of cells making up the hypocotyl are colonised whereas at 7 DPI no apparent cellular invasion events can be observed with this method (Figure 1c). The visibility of Nile Red staining depends on the type of object and applied microscopy technique, in our case we used it only for broad tissue inspection to see when the disease progression and plant colonisation becomes evident. Such staining is strongest in late infection time points, when *P. brassicae* resting spores are produced (Figure 1c, 26 DPI).

To compare local versus systemic reactions, samples were taken from hypocotyls (belowground parts colonised by *P. brassicae*) and petioles of the fully expanded first true leaves (source organs potentially involved in the redistribution of nutrients). Groups of phloem cells comprising phloem parenchyma (PP), companion cells (CC) and sieve elements (SE) were isolated from paraplast‐embedded sections with a laser dissecting microscope (Figure 1d). Amplified total RNA isolated from dissected tissue was used for the preparation of cDNA and sequencing libraries for transcriptomic analyses. Although, at 7 DPI, staining showed that there was not yet a strong build‐up of the pathogen, there were 1473 differentially expressed genes (DEGs fold change ≥2, FDR ≤0.05)—1100 upregulated and 373 downregulated, in comparison to mock‐inoculated plants in the hypocotyl phloem (Figure 1e). At 12 DPI infection impacted a larger number of genes, and differential accumulation of 8124 transcripts was observed, 5367 upregulated and 2757 downregulated (Figure 1e). Concomitantly, we observed significant number of gene reads mapping to the *P. brassicae* genome only in the phloem samples isolated from hypocotyls at 12 DPI (Supplementary File 1) and the comparison of the transcriptomic profiles showed strikingly distinct expression profiles at this time point (Figure 1g). Altogether this reflects the depth of hypocotyl colonisation (Figure 1c), further disease progression and hijacking of the host physiology and development by the pathogen. The number of DEGs sharing responses across time points in the same sample category (hypocotyl or petiole) was relatively small and did not exceed 3% of total DEGs (1.81% in hypocotyls and 2.05% in petioles) which also shows the shifting impact of disease progression across time (Figure S1). Transcriptional changes in phloem cells isolated from petioles affected fewer genes than in hypocotyls (only 327 and 421 differentially expressed genes at 7 and 12 DPI, respectively) (Figure 1e,f). This reflects the fact that *P. brassicae* does not colonise the aboveground parts of the host, so only systemic responses occur here. In previous work carried out by Irani et al. (2018), a higher number of DEGs in aerial tissue in comparison to the galls of *P. brassicae* infected *A. thaliana* was observed. This discrepancy might be related to the fact that later time points and whole plant organs were analysed. Comparison of similarities between aboveground and belowground phloem samples in our dataset shows that only 219 out of the 730 DEGs identified in petioles were also differentially expressed in hypocotyls of *P. brassicae* infected OSR plants (Figure S1). This could reflect fundamental differences between environments and distinct functional aspects of phloem‐mediated responses. This was further reflected in the clear separation of transcriptional profiles between the hypocotyl and petiole phloem samples (Figure 1g). For the initial assessment of the functional character of observed transcriptional changes a Gene Ontology (https://geneontology.org/) enrichment analysis was performed (Supplementary File 1). We visualised increases in selected enriched GO term categories and found that defence, wound and hormonal responses dominate both at 7 and 12 DPI in phloem from hypocotyls and petioles (Figure S2a–d). The comparison of GO terms enriched within upregulated genes (Benjamini–Hochberg [BH] adjusted *P*‐value <0.05) in petioles at 7 and 12 DPI (Figure S2c,d) shows an over‐representation of genes associated with the response to jasmonic acid (JA), wounding, reactive oxygen species, biotic stimulus and growth regulators. At 12 DPI in phloem from petioles (Figure S2d) additionally enrichment of GO term categories related to the regulation of nitrogen compound metabolic process was observed. In phloem samples isolated from galls at 7 DPI, enrichment of GO term categories related to both defence, hormonal regulation and changes in carbon and nitrogen economy was observed already (Figure S2a). At 12 DPI, even stronger enrichment in response to the hormone category as well as a strong increase in regulation of nitrogen compound metabolic process was seen (Figure S2b).

Taken together, this analysis depicts the relation between phloem‐specific host responses and the predicted role of this tissue in either the pathogen‐driven sink or the distal source organs. The disruption of the phloem differentiation process in Arabidopsis caused accelerated death of *P. brassicae*‐infected plants (Walerowski et al., 2018). When we attempted to block phloem transport by inducible callose deposition in sieve elements, using previously characterised *pAPL::XVE>>icals3m* line (Vatén et al., 2011), it led to a decrease in gall size and overall exacerbated disease symptoms (Figure S3). This further supports the role of this tissue in coordinating post‐infectious host responses to *P. brassicae*. Not surprisingly, we can observe transcriptional changes in phloem that are involved in the coordination of numerous aspects of plant function but the intensity and degree of reprogramming will differ between two environments (belowground and aerial) and correlate with the local presence of the pathogen.

### Transcriptomic profiling of *B. napus* phloem cells highlights responses characteristic of compatible plant–microbe interactions

**Figure 2:**
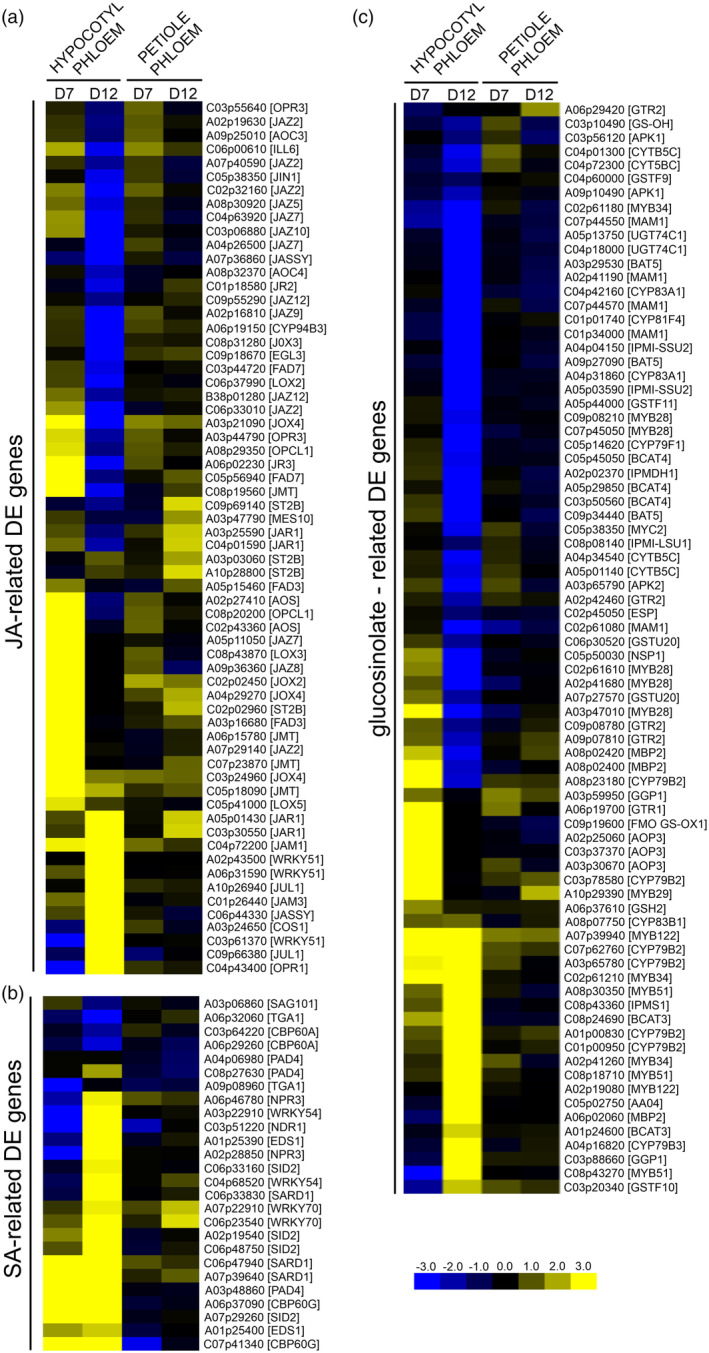
Transcriptional profiling of *Brassica napus* phloem tissue shows local and long‐distance modulation of plant defence response to *Plasmodiophora brassicae* colonisation. (a–c) Heatmaps of log_2_ fold change values of the identified DEGs in the hypocotyl and petiole phloem samples from the *P. brassicae‐*infected plants 7 and 12 DPI involved in plant defence responses, including (a) JA‐related DEGs, (b) SA‐related DEGs and (c) glucosinolate‐related DEGs. Upregulated genes are shaded in yellow and downregulated genes in blue. Significant DEGs were identified based on a log_2_ ratio ≥1 and a false discovery rate ≤0.05.

**Figure 3:**
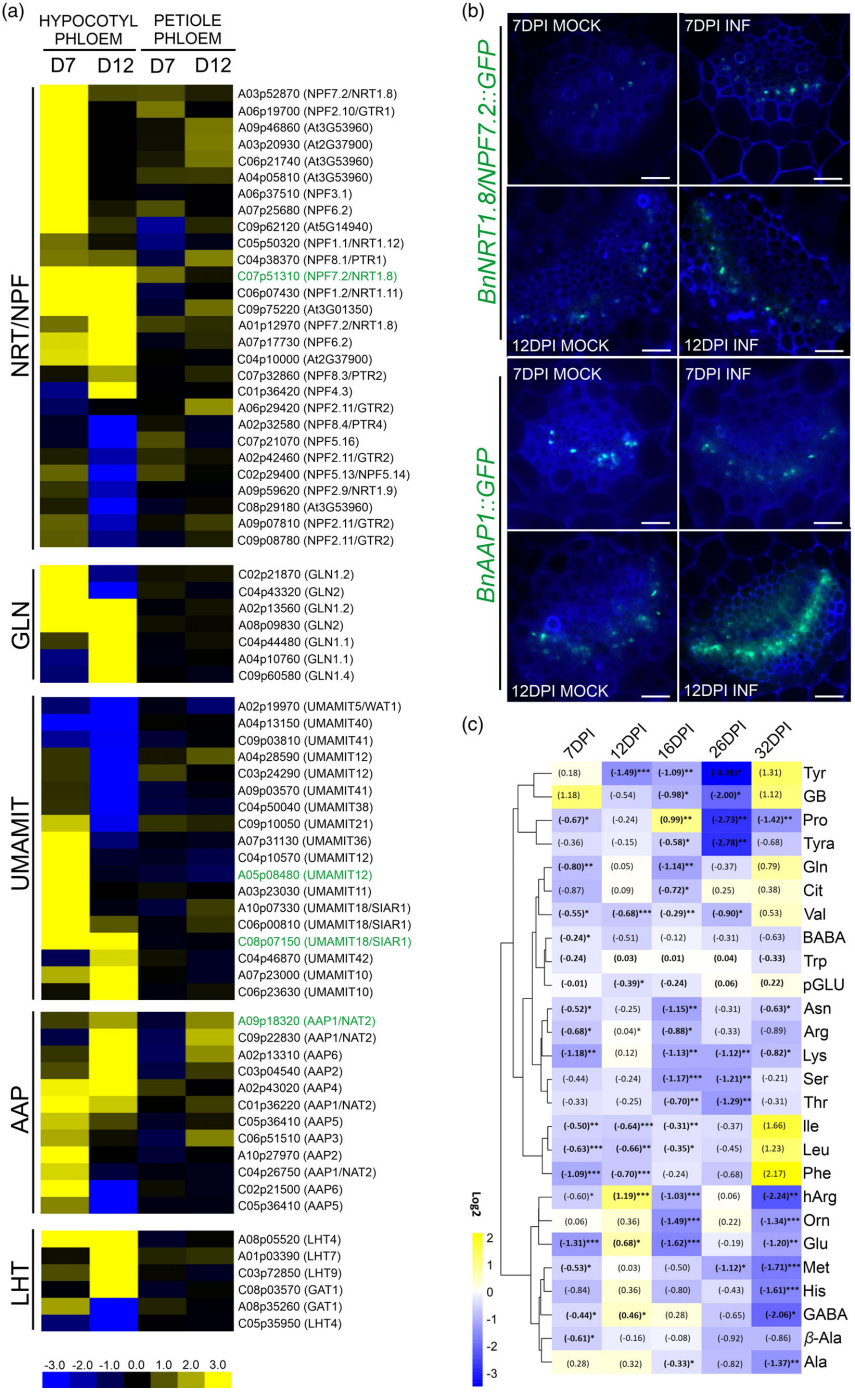
Pathogen hijacks the internal host mechanisms for nitrogen‐rich components repartitioning. (a) Heatmaps of log_2_ fold change values of the identified DEGs in the hypocotyl and petiole phloem samples from the *Plasmodiophora brassicae‐*infected *Brassica napus* plants 7 and 12 DPI involved in nitrogen‐related responses, including nitrate transporters (NRT/NPF), glutamine synthetase encoding genes (GLN), Usually Multiple Acids Move In and out Transporters (UMAMIT), amino acid permeases (AAP) and amino acid transporters Lysine/Histidine‐like Transporter (LHT). Upregulated genes are shaded in yellow and downregulated genes in blue. Significant DEGs were identified based on a log_2_ ratio ≥1 and a false discovery rate ≤0.05. (b) Promoter activities of nitrate transporter *NRT1.8*/*NPF7.2* and AA transporter *AAP1* orthologues visualised at 7 and 12 DPI in transverse sections of petioles from mock‐treated and *P. brassicae‐*infected Arabidopsis plants, using promoter::GFP reporter system and Calcofluor white as a counterstain. Scale bars represent 50 μm. (c) Heatmap of log_2_ fold change values of the detected AAs in the phloem exudates from the leaves of *P. brassicae*‐infected *B. napus* plants at 7, 12, 16, 26 and 32 DPI relative to the content in mock‐treated plants, with five independent biological replicates, each containing 60 leaves (3 leaves from 20 plants). Upregulated genes are shaded in yellow and downregulated genes in blue. The fold change values are shown in parenthesis, and asterisks indicating statistically significant differences where *P ≤ 0.05, **P ≤ 0.01 and ***P ≤ 0.001 are provided as superscript. For data that shows a normal distribution an unpaired *t*‐test was performed whereas for non‐normal distribution a Wilcoxon signed‐rank test was used.

In phloem dissected from *B. napus* hypocotyls, we observed enrichment of JA‐related transcripts at 7 DPI (Figure 2a). Previous surveys in which whole galls were used for the analysis also showed induction of JA‐mediated signalling (Aigu et al., 2022). In our experiment, induction of the JA‐responsive genes was also observed in phloem cells isolated from petioles both at 7 and 12 DPI. This is in agreement with the previously described systemic character of JA signalling (Parker, 2009). Interestingly, at 12 DPI, numerous JA‐related gene transcripts such as jasmonate ZIM‐domain (*JAZ*) repressors of JA‐regulated gene transcription, JA‐induced oxygenases (*JOX*) involved in JA catabolism and also JA biosynthesis‐related genes including *AOC* and *JASSY* involved in the transport of OPDA precursors were significantly lower (Figure 2a). On the other hand, transcripts of *WRKY51* and *WRKY70* orthologues, known in Arabidopsis to be involved in the salicylic acid (SA)‐induced suppression of JA pathways and the regulation of antagonistic crosstalk between JA and SA (Li et al., 2006; Pieterse et al., 2012), were elevated in the hypocotyl‐derived phloem at 12 DPI. At this time point, numerous SA‐response genes are also upregulated in phloem in infected hypocotyls (Figure 2b). Differences between these two time points (7 and 12 DPI) could also be observed for glucosinolate‐related gene transcripts (Figure 2c). In this case, at 12 DPI, we observed decreased levels of numerous Arabidopsis orthologue gene transcripts whose products were previously found to be involved in aliphatic glucosinolate synthesis (*MAM1*, *BCAT*, *BAT* and *CYP79F1*). At the same time, indolic glucosinolate biosynthesis and regulation‐related gene transcripts (*CYP79B2/3*, *CYP83B1*, *MYB34*, *MYB51* and *MYB122*) were upregulated. It has been reported that indolic glucosinolates may be used as a substrate for auxin biosynthesis, therefore observed increases could be related to *P. brassicae*‐induced gall formation (Ludwig‐Müller, 2009). The observed drop in the JA‐ and aliphatic glucosinolate‐related transcripts at 12 DPI may reflect the suppression of host defence that is necessary for hypocotyl colonisation and formation of the pathogen niche. The difference in local responses in hypocotyl phloem tissue between 7 and 12 DPI was further visible in the expression pattern of high‐affinity plasma membrane‐localised glucosinolate (GLS) importers *GTR1/NPF2.10* and *GTR2/NPF2.11* whose transcript levels increased at 7 but not at 12 DPI (Figures 2c and 3a). The GTR transporters are involved in glucosinolate import from the apoplastic space to phloem and play a role in the chemical protection of this conduit from colonisation and systemic spread of the pathogen (Hunziker et al., 2019; Xu et al., 2019).

The interplay between JA and SA as well as various GLS responses in clubroot‐infected plants were studied previously and our findings are in accordance with previous reports (Galindo‐González et al., 2020; Lemarié et al., 2015; Xu et al., 2018). On the other hand, we had an opportunity to compare patterns observed in non‐colonised versus colonised plant parts and this shows that in susceptible hosts, *P. brassicae* suppresses wound‐related responses at the infection site, but not in the non‐colonised part (phloem isolated from leaf petioles). We hypothesise that this could be an important pathogen‐driven modification of the host response that allows it to build up a physiological sink. It does not block the host systemic post‐infection signalling necessary for other developmental adjustments mediated *via* phloem in *P. brassicae*‐infected susceptible plants.

### Transcriptomic profile of phloem dissected cells indicates carbon and nitrogen redistribution along the vasculature during the early stages of clubroot disease

Gazengel et al. (2021) have shown that altering the supply of nitrogen can shape the dynamics of clubroot disease progression. In addition, Aigu et al. (2022) have demonstrated that this aspect may, to some extent influence the degree of tolerance to clubroot in *B. napus*. This can be related to the ability of the invaded host to relocate and ‘invest’ nutrients to shape growth responses or restrict their availability to the pathogen. The upregulation of particular transcripts that we observed in phloem tissue seems to support the role of the phloem in this mechanism. Our GO term analysis indicated the possible involvement of both hypocotyl and petiole‐derived phloem cells in nitrogen repartitioning (Figure S2a–d). In agreement with this, we have observed differential expression of numerous transcripts whose products may influence nitrogen economy in host plants. Firstly, at 7 and 12 DPI, we can observe increased expression of Arabidopsis *NPF7.2*/*NRT1.8* nitrate transporter orthologues *A03p52870.1_BnaDAR*; *C07p51310.1_BnaDAR* and *A01p12970.1_BnaDAR* (Figure 3a). In Arabidopsis, it has been shown that this nitrate influx transporter may be involved in the JA/ET induced nitrate relocation to roots to mediate growth trade‐offs related to stress responses (Li et al., 2010; Zhang et al., 2014a). We speculate that in clubroot infected plants its expression is hijacked by *P. brassicae* to strengthen its own sink.

The nitrogen remobilisation was further reflected by the upregulation at 7 DPI (both hypocotyl and petiole) of *C07p25680.1_BnaDAR* and at 7 and 12 DPI of *A07p17730.1_BnaDAR*. These genes are orthologues of the Arabidopsis low‐affinity nitrate transporter *NPF6.2* involved in both N and K flux mediation (Morales de los Ríos et al., 2021). We also observed differential expression of *C06p07430.1_BnaDAR* and *C05p50320.1_BnaDAR*, orthologues of the *NRT1.11* and *NRT1.12* transporters from Arabidopsis. These low‐affinity nitrate transporter's expression was previously localised in the phloem companion cells of the fully expanded leaf midvein and their role was assigned to the xylem to phloem redistribution of root‐derived nitrate from fully expanded (source) to young (sink) leaves (Hsu & Tsay, 2013). In our dataset, *NRT1.11* and *NRT1.12* are downregulated in phloem isolated from petioles of fully expanded leaves at 7 DPI whilst *NRT1.11* is upregulated in phloem from hypocotyls both at 7 and 12 DPI. This situation may reflect that clubroot‐infected plants cannot afford stimulation of leaf growth by nitrate redirection to young sink leaves and in fact the newly emerging pathogen‐oriented sink within the phloem outcompetes host organs. It has been commonly observed that clubroot infection is accompanied by a decrease in leaf growth whereas enhanced hypocotyl secondary thickening may actively consume nitrates in clubroot galls.

Another group of transporters that are known to be involved in source–sink remobilisation are amino acid permeases (AAPs). We found that the orthologues of Arabidopsis *AAP1*, *AAP2*, *AAP5* and *AAP6* are differentially regulated in phloem within hypocotyls of clubroot‐infected *B. napus* (Figure 3a). Transcripts of the Arabidopsis *AAP1* and *3* orthologues were also significantly upregulated in phloem cells dissected from petioles, however, this change has been observed only at 12 DPI (Figure 3a). Previously *AAP1*, *AAP2* and *AAP6* were found to be involved in the xylem to phloem AA import, supporting in this way the development of sink organs in various plant species (Hunt et al., 2009; Zhang et al., 2010, 2015).

In phloem dissected from hypocotyls, we also observed increased levels of the high‐affinity amino acid transporters lysine/histidine‐like transporter orthologues *LHT7* and *LHT4* (Figure 3a). Members of this family may be involved in loading various amino acids from extracellular space into the phloem (Tegeder & Ward, 2012).

We also found elevated levels of the Arabidopsis Usually Multiple Acids Move In and out Transporters (UMAMIT) orthologues in our *B. napus* phloem transcriptome dataset (Figure 3a). The *UMAMIT36* orthologue was upregulated at 7 DPI whereas *UMAMIT 18* and *42* were upregulated at 12 DPI. Some of the UMAMITs were significantly downregulated at 12 DPI (*UMAMIT5*, *12*, *21*, *38*, *40* and *41*). So far, the involvement of UMAMIT18 in amino acid delivery to developing seeds has been reported (Ladwig et al., 2012). We can speculate that the increased expression in hypocotyls (future site of gall formation) may be related to the unloading of AAs from the phloem to support the developing pathogen sink, however, this needs further functional studies. It was proposed that in leaves some members of the UMAMIT transporter family may be involved in amino acid transfer from the phloem parenchyma into the apoplast (Kim et al., 2021). The only significantly differentially expressed UMAMIT orthologue in phloem from petioles was *A02p19970.1_BnaDAR* (*UMAMIT5*) and its levels in fact decreased (Figure 3a).

At 12 DPI in hypocotyl phloem, we also found increased expression of the *AMT2.2* ammonium transporter orthologue. Recent work of Giehl et al. (2017) shows that AMT2.1 is involved in root‐to‐shoot ammonia translocation. Apart from that, in phloem dissected from hypocotyls of infected plants, we have observed elevated transcript levels of the *B. napus* orthologues of Arabidopsis glutamine synthetase encoding genes (*GLN*s) (Figure 3a), whose action has been previously linked to ammonium assimilation (Konishi et al., 2016).

To check cellular transcript accumulation patterns, we decided to clone the 1.5 kb promoter regions of selected differentially expressed nitrate and AA transporter genes from OSR, fusing them to the GFP coding sequence to observe promoter activity in Arabidopsis plants. We were able to clone *NPF7.2* (*C07p51310.1_BnaDAR*), *UMAMIT12* (*A05p08480.1_BnaDAR*), *UMAMIT18* (*C08p07150.1_BnaDAR*) and *AAP1* (*A09p18320.1_BnaDAR*). Comparison of cloned sequences against the same promoter regions from the closest orthologues from Arabidopsis has shown 44.6%, 44.6%; 57.2% and 46.4% identity, respectively. For all four genes, we were able to confirm phloem‐localised expression in petioles from fully expanded Arabidopsis leaves regardless of *P. brassicae* infection (Figure 3b; Figure S4a,b). Interestingly, in the case of two tested promoters, we were able to observe induction in response to *P. brassicae* infection. For *NPF7.2* (*C07p51310.1_BnaDAR*) stronger GFP signals were observed upon infection both at 7 and 12 DPI whereas for *AAP1* (*A09p18320.1_BnaDAR*) increase upon infection was observed at 12 DPI (Figure 3b). For the tested UMAMITs no apparent differences in the GFP signals were visible (Figure S4a,b).

To gain insight into the possible redirection of free amino acids, phloem exudates from fully expanded leaves of mock‐ and *P. brassicae*‐inoculated *B. napus* plants were collected and subjected to mass spectrometry analysis. A decrease in levels of most AAs (Figure 3c; Table 1) were observed upon infection. The only significant increases were detected for glutamic acid (Glu), γ‐aminobutyric acid (GABA) and homoarginine (hArg) at 12 DPI, and later at 16 DPI, higher levels of proline (Pro) were observed (Figure 3c; Table 1). For these compounds, the accumulation pattern also differed between inoculated and control combinations across the time, whereas other AAs followed fluctuations in the controls but just at lower levels (Figure 3c; Table 1).

**Table 1:** Changes in the content of amino acids observed in phloem sap of oilseed rape plants upon *Plasmodiophora brassicae* infection

Free AAs (mean ± SD)	7 DPI				12 DPI				16 DPI				26 DPI				32 DPI			
	Non‐infected		Infected		Non‐infected		Infected		Non‐infected		Infected		Non‐infected		Infected		Non‐infected		Infected	
Ala	1495.43	130.38	1820.01	624.84	435.59	102.41	545.81	66.26	413.99	54.80	328.55	18.57	387.59	137.79	219.15	145.08	436.52	62.96	168.86	137.59
Arg	10 778.78	3519.00	6711.39	1284.99	3299.22	471.77	3394.11	1967.12	6754.21	1596.08	3675.94	1331.70	3887.98	954.32	3094.50	1157.44	3227.37	719.76	1740.28	764.29
Asn	8405.08	1833.21	5869.36	1097.58	2989.46	419.27	2510.37	273.82	4875.04	1626.88	2194.45	373.50	1060.58	146.90	853.93	214.39	744.62	108.79	479.57	217.91
BABA	152.77	20.46	129.21	29.20	195.47	24.47	136.34	14.71	333.57	31.98	305.68	32.90	206.84	64.30	163.10	89.61	258.58	59.32	189.21	106.56
B‐Ala	36.72	5.47	24.04	9.95	16.36	3.60	14.56	2.87	6.66	1.11	6.30	1.87	4.16	2.39	2.20	1.40	3.08	1.13	2.12	0.33
Cit	22.67	8.50	12.37	1.03	4.22	1.72	4.48	1.06	4.31	1.33	2.61	0.43	3.22	0.58	3.84	1.45	2.49	0.38	3.23	1.18
GABA	47 329.65	7090.70	34 899.16	5388.51	22 821.47	4835.79	31 429.09	6761.01	20 914.25	4173.36	25 462.90	4520.01	33 266.68	11 146.24	21 162.97	14 323.75	49 592.62	2759.42	14 847.95	1504.61
GB	0.33	0.17	0.74	0.54	0.60	0.17	0.41	0.20	2.88	0.97	1.46	0.65	2.49	1.53	0.62	0.17	0.65	0.20	1.41	0.77
Gln	78 072.89	17 139.03	44 751.18	10 906.67	41 269.74	6231.24	42 853.52	14 463.62	97 399.02	25 813.94	43 939.36	7964.30	21 046.10	4549.60	16 235.80	4350.47	11 110.33	1465.40	19 162.88	11 168.92
Glu	404 43.57	9365.24	16 354.56	2133.26	9973.63	1272.34	15 998.21	4074.39	13 875.30	1026.15	4512.91	976.62	1670.43	221.56	1459.68	342.84	906.79	105.86	393.67	197.58
hArg	741.16	178.68	488.71	59.15	84.02	19.73	191.56	17.62	305.22	45.73	149.01	29.54	170.39	85.34	178.17	33.55	339.55	170.13	71.61	8.79
His	84.48	35.45	47.11	14.36	36.93	8.67	47.30	17.93	104.16	25.76	59.88	21.05	80.84	23.37	60.19	31.45	85.80	16.32	28.11	3.62
Ile	20 141.05	2450.51	14 252.84	2615.16	22 134.72	1652.67	14 238.39	1033.20	24 685.92	1951.89	19 899.21	1221.56	12 571.23	2783.85	9711.14	4920.29	3879.12	514.97	12 280.71	7066.12
Leu	6085.60	280.37	3929.92	575.31	8378.42	955.96	5290.27	1031.32	14 200.84	1288.83	11 140.85	2532.24	6839.09	1951.35	4999.61	2069.95	2610.34	422.73	6118.85	3480.52
Lys	315.44	92.19	140.10	21.95	119.97	53.23	130.16	40.98	586.73	191.49	267.98	71.72	116.09	34.61	53.30	11.80	71.19	21.15	40.38	7.64
Met	11.99	1.99	8.28	2.66	6.26	1.20	6.39	2.32	15.55	5.29	11.00	1.41	30.42	9.50	14.02	11.84	39.24	4.17	11.99	5.91
Orn	746.60	45.56	776.98	105.85	499.05	175.87	642.46	149.12	1665.60	177.53	591.80	219.96	765.07	143.24	890.32	226.42	1036.67	146.19	408.58	167.46
pGlu	219.59	13.33	217.76	47.54	87.19	13.86	66.67	8.32	147.05	42.92	124.12	47.35	130.22	19.61	136.51	15.85	153.11	26.81	178.04	100.00
phe	14 428.43	1242.26	6776.88	2335.18	23 621.78	1847.32	14 509.18	2298.54	30 768.20	4198.25	26 025.76	3690.04	16 107.79	4638.03	10 036.89	4501.19	3903.43	528.49	17 643.37	10 311.61
Pro	12 131.02	544.88	7641.21	2986.55	5647.17	612.03	4770.54	1255.41	8321.70	2646.44	16 530.12	4001.58	12 682.63	6089.22	1906.53	646.12	3899.27	480.48	1455.97	826.33
Ser	2162.98	477.14	1589.84	340.87	1963.79	267.97	1653.24	276.47	3652.66	668.70	1623.19	308.96	1341.36	578.67	580.45	175.82	560.39	156.83	483.24	209.66
Thr	20.57	1.87	16.40	5.35	19.99	4.40	16.78	2.96	30.68	6.95	18.95	2.50	16.27	5.95	6.66	1.62	7.29	0.89	5.87	2.87
Trp	29 041.94	2124.13	24 592.11	6472.42	23 345.16	1247.75	23 845.07	7621.43	17 965.40	3002.08	18 161.26	1339.99	19 588.56	3917.17	20 152.27	2267.45	23 628.77	4812.15	18 798.53	7904.39
Tyr	200.73	26.92	227.15	98.19	416.21	39.21	147.90	26.24	1978.18	493.91	926.45	225.95	628.29	385.18	56.09	5.51	40.17	10.14	99.80	53.28
Tyra	646.70	223.25	503.03	192.17	455.81	220.23	409.70	108.30	652.02	176.47	435.52	98.90	23.08	16.76	3.35	1.58	4.79	1.90	2.97	1.41
Val	19 380.96	1838.27	13 195.55	3720.72	23 352.75	2270.72	14 544.24	2309.41	28 615.14	2803.14	23 442.21	1954.56	17 065.29	5404.66	9170.42	3200.11	7617.71	1123.17	11 015.50	6610.12

The mean values for five independent biological replicates are presented along with SD.

**Figure 4:**
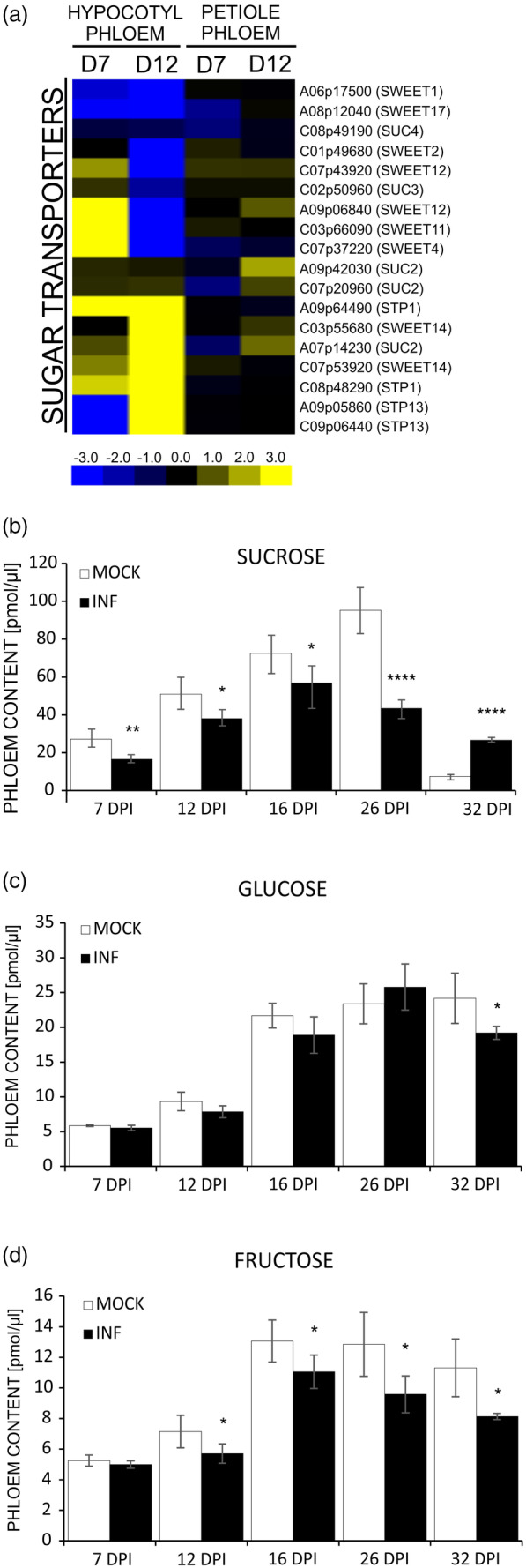
Clubroot disease progression is accompanied by differential expression of sugar transporters, however, the concentration of sucrose in the phloem sap of *Plasmodiophora brassicae*‐infected *Brassica napus* plant decreases. (a) Heatmap of log_2_ fold change values of the identified differentially expressed sugar transporter genes in the hypocotyl and petiole phloem samples from the *P. brassicae‐*infected plants at 7 and 12 DPI. Upregulated genes are shaded in yellow and downregulated genes in blue. Significantly differentially expressed genes were identified based on a log_2_ ratio ≥1 and a false discovery rate ≤0.05. (b–d) Content of (b) sucrose, (c) glucose and (d) fructose in the phloem exudates from the leaves of mock‐treated and *P. brassicae*‐infected *B. napus* plants measured at 7, 12, 16, 26 and 32 DPI with five independent biological replicates, each containing exudates collected from 60 leaves (3 leaves from 20 plants). Asterisks indicate statistically significant differences at *P ≤ 0.05, **P ≤ 0.01, ***P ≤ 0.001, ****P ≤ 0.0001, according to unpaired *t*‐test for data showing normal distribution or by Wilcoxon signed‐rank test for data with non‐normal distribution.

Another group of nutrients transported *via* phloem are carbohydrates. It is already known that *P. brassicae* leads to the formation of a strong carbohydrate sink and developing galls are sites of intense carbohydrate consumption (Evans & Scholes, 1995; Walerowski et al., 2018). In our dataset, we found differential expression of carbohydrate transporter genes (Figure 4). Transcriptional profiling in phloem dissected cells from oilseed rape hypocotyls at 7 DPI shows increased levels of the *SWEET4* and *SWEET11* orthologues. Strong positive fold change but a marginal *P*‐value (0.052) was also seen for transcripts of a *SWEET12* orthologue. At 12 DPI, we mainly observed decreases (*SWEET1,2,4,11,12,17*) and only expression of *SWEET14* orthologues were highly upregulated in comparison to mock‐inoculated samples (Figure 4a). Furthermore, DEGs encoding *SUGAR TRANSPORTER PROTEIN* (*STP*) orthologues were amongst those significant in hypocotyl phloem (at 12 DPI, two *STP1* and two *STP 13* orthologues were upregulated). In phloem cells isolated from petioles, changes were less pronounced and in fact none of the *SWEET* transporter genes expression differed significantly. At 7 DPI, we could see decreased levels of *SUC2* and *SUC4* orthologues whereas at 12 DPI, *SUC2* transcripts were elevated. No significant change in the *STP* transporters expression was observed in phloem cells dissected from petioles.

Further examination of sucrose, glucose and fructose levels in phloem exudates isolated from fully expanded OSR leaves of mock‐inoculated and *P. brassicae* infected plants at 7, 12, 16, 26 and 32 DPI showed that, despite differential accumulation of carbohydrate transporter transcripts, sucrose levels in phloem drops already from the very early stages of disease (Figure 4b). The concentration of this component increases only at the time when resting spores reach maturity (32 DPI). Apart from 32 DPI where significant decreases became evident for glucose, its levels remained unaltered throughout (Figure 4c). A decrease in fructose levels started from 12 DPI and this trend continued through progressive stages of disease progression (Figure 4d).

Our LCM‐assisted phloem transcriptomics shows complex reprogramming of AAs, nitrate and sugar transporters accompanying pathogen sink formation and maintenance during the time of decreased host nutrient assimilation (Figures 3a and 4a). As a consequence of clubroot disease progression, the availability of carbon‐ and nitrogen‐rich components in OSR plants might decrease. This is supported by the decreases in photosynthesis, CO_2_ assimilation and transpiration (Figure S5) which results in a situation where the infected host suffers from nutrient deficits. This does not change the fact that the pathogen continues to starve the host to support its own nutrient demands. Despite the fact that the content of nitrogen‐ and carbon‐rich components transported over long distances decreases (Figures 3c and 4b–d), our transcriptomic data presents a pattern suggesting significant reshuffling of nitrates, AAs and sugars at the level of phloem tissue. Differential expression can be observed both at the pathogen site (hypocotyls) or systemically in non‐colonised regions (petiole phloem). At present based on transcriptomic fingerprints and metabolic studies we cannot assign particular physiological roles in the translocation of nitrogen‐ and carbon‐rich components to individual factors, however, the obtained results can be used as a foundation for future functional studies.

### Cytokinin‐mediated host developmental responses are modulated *via* phloem during clubroot disease

**Figure 5:**
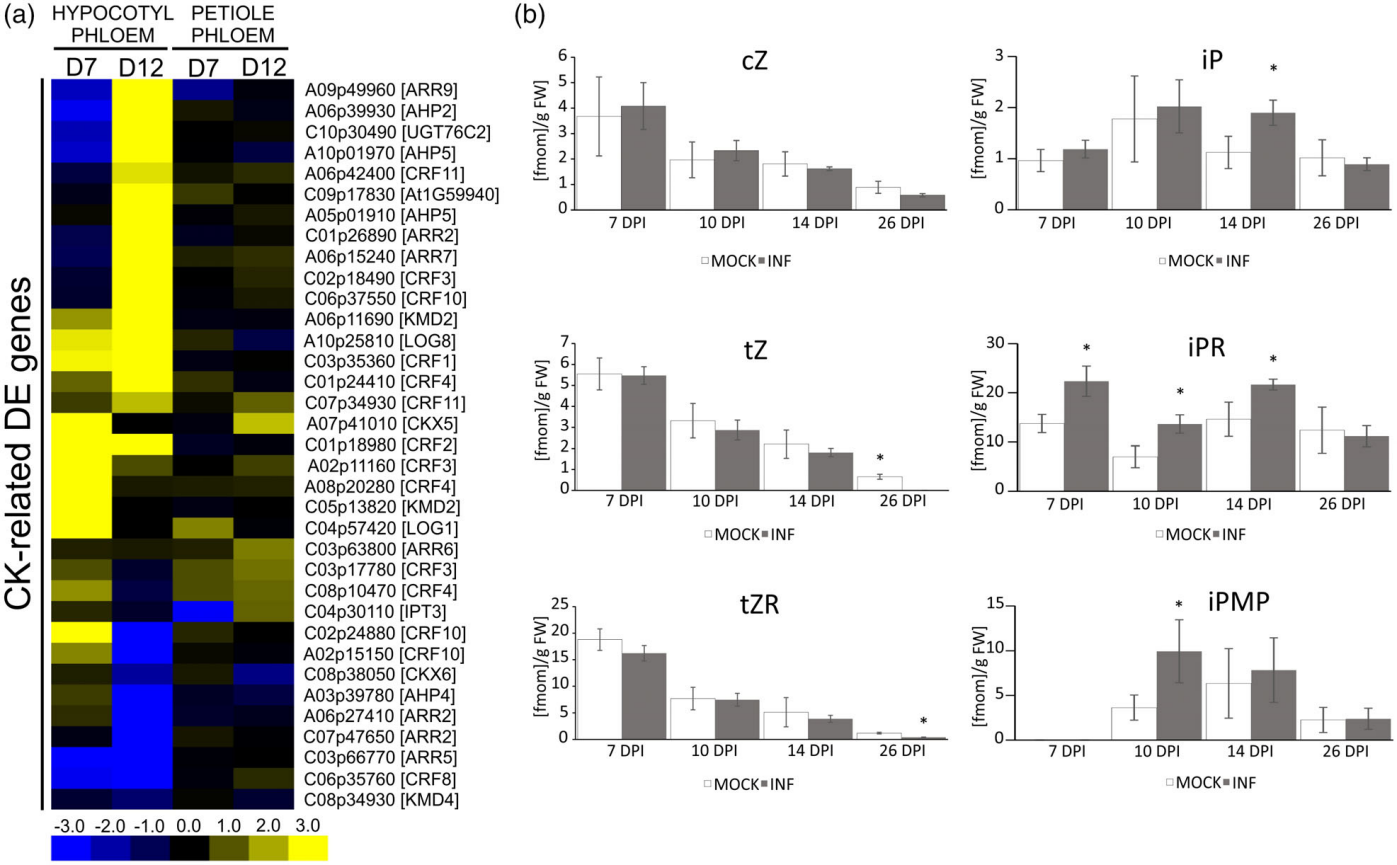
Transcriptional changes in cytokinin‐related factors as well as elevated levels of the iP type cytokinins in phloem sap suggest the role of these hormones in long‐distance coordination of host responses to clubroot disease. (a) Heatmaps of log_2_ fold change values of the identified DEGs in the hypocotyl and petiole phloem samples from the *Plasmodiophora brassicae‐*infected plants 7 and 12 DPI related to cytokinin (CK) signalling. Upregulated genes are shaded in yellow and downregulated genes in blue. Significantly differentially expressed genes were identified based on a log_2_ ratio ≥1 and a false discovery rate ≤0.05. (b) Content of different cytokinin forms, including cis‐Zeatin (cZ), trans‐Zeatin (tZ), trans‐Zeatin Riboside (tZR), *isoPentenyladenine* (iP), *isoPentenyladenine* Riboside (iPR) and isoPentenyladenosine‐5′‐MonoPhosphate (iPMP) in the phloem exudates from the leaves of mock‐treated and *P. brassicae*‐infected *Brassica napus* plants measured at 7, 10, 14 and 26 DPI, with five independent biological replicates, each containing exudates collected from 60 leaves. Asterisks indicate statistically significant differences at *P* ≤ 0.05 that were calculated using unpaired *t*‐test for results that showed normal distribution, or by Wilcoxon signed‐rank test for data that did not follow a normal distribution.

In phloem tissue dissected from infected *B. napus* plants (both hypocotyl and petiole), numerous cytokinin response gene transcripts were differentially expressed (Figure 5a). Amongst them, a major group that showed stimulated transcriptional response contained orthologues of *CYTOKININ RESPONSE FACTORS* (*CRFs*). In Arabidopsis, the action of *CRF3* and *4* was previously linked to the coordination of cytokinin signalling with auxin developmental responses during plant organ growth (Raines et al., 2016) and numerous reports show CRFs involvement in the coordination of nutrient‐dependent organ growth feedbacks in plants (Sakakibara, 2021). Cytokinin synthesis takes place in the phloem, and then they are subsequently loaded to the xylem and transported with the help of the ABCG14 transporter to the upper parts of plants, where these phytohormones are redistributed *via* the phloem to regulate organ growth (Ko et al., 2014; Sakakibara, 2021; Zhang, Novak, et al., 2014; Zhao et al., 2021). It has been suggested that this xylem‐mediated translocation of cytokinins may also be an important long‐distance signal for nitrate deprivation or increased nitrate demand (Sakakibara, 2021).

To further test the possible role of phloem in cytokinin redistribution during clubroot disease, we examined *B. napus* exudates. We found that decreases in aboveground plant growth, already visible during the proliferative stage of gall formation, are accompanied by increases in the active (iP), transport (iPR) and primary biosynthetic (iPMP) forms of isopentenyl‐type cytokinins in *B. napus* phloem sap (Figure 5b). The iP level was significantly higher at 14 DPI, the iPR at 7, 10 and 14 DPI, whereas the iPMP increased at 10 DPI. We hypothesise that amongst these, the iPR transport form may play an important role in long‐distance phloem‐mediated regulation of host developmental responses to *P. brassicae* infection. Previous works suggest that phloem‐mediated basipetal transport of the iP‐type cytokinins may be involved in the orchestration of vascular patterning in Arabidopsis roots (Bishopp et al., 2011; Vatén et al., 2011), and we know that both phloem and xylem differentiation is altered in developing galls of both OSR and Arabidopsis plants (Gustafsson et al., 1986; Malinowski et al., 2012).

**Figure 6:**
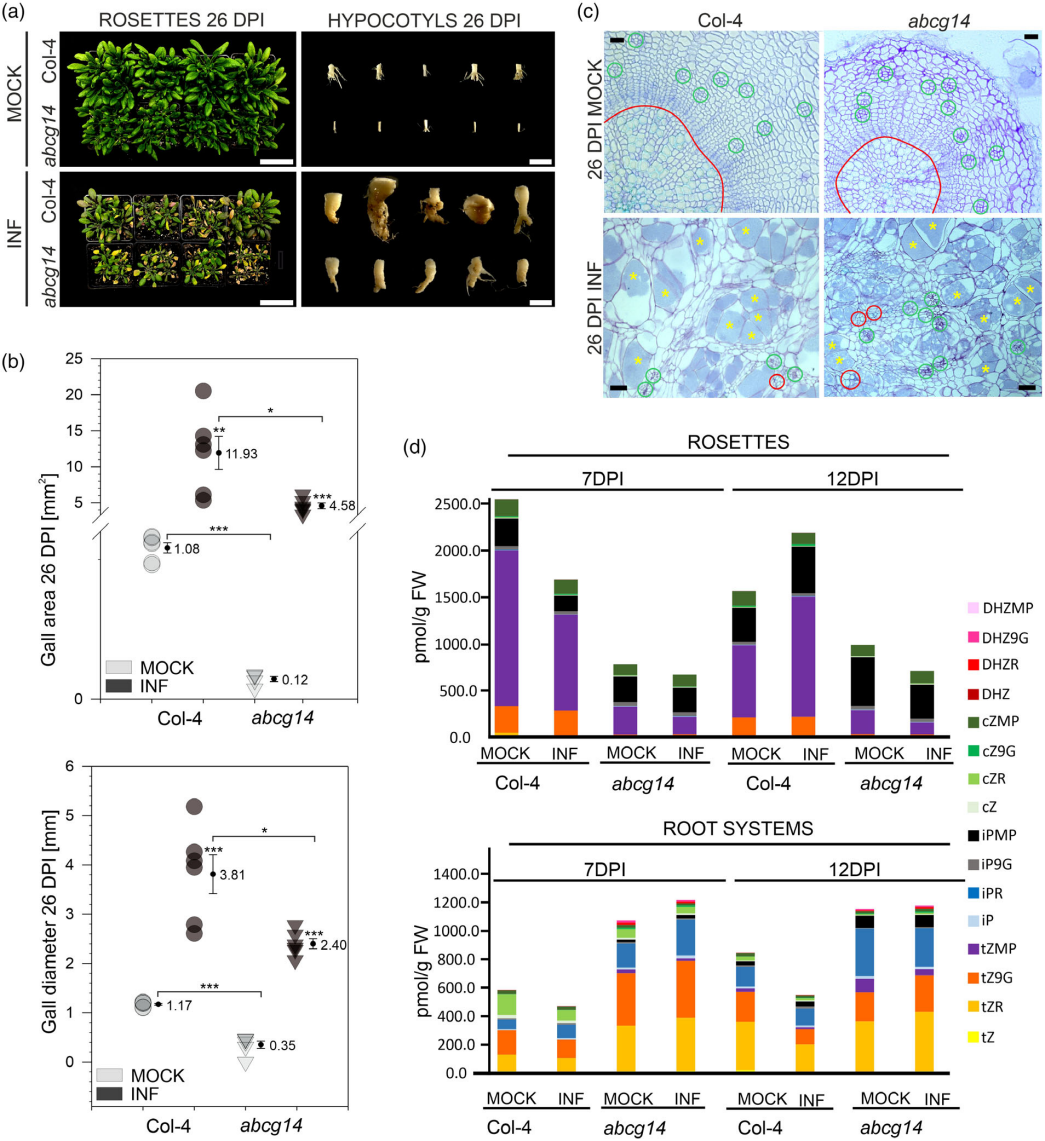
Disruption of cytokinin transport affects growth responses without impacting *Plasmodiophora brassicae* lifecycle progression in the infected host. (a) Clubroot disease symptoms in the rosettes and hypocotyls of Arabidopsis Col‐4 and mutant *abcg14* plants 26 DPI. Scale bars represent 5 cm and 5 mm for rosettes and hypocotyls, respectively. (b) Changes in hypocotyl area and diameter were observed between *Atabcg14* mutant and Col‐4 mock‐inoculated and *P. brassicae*‐infected plants. Calculated means and SEs are presented. Asterisks indicate statistically significant differences between means at *P ≤ 0.05, **P ≤ 0.01, ***P ≤ 0.001, according to the unpaired *t*‐test. (c) Hypocotyl cross sections of mock‐treated and *P. brassicae‐*infected wild‐type Col‐4 and *Atabcg14* plants 26 DPI stained with toluidine blue. Scale bars represent 20 μm for mock‐inoculated and 50 μm for *P. brassicae*‐infected radial hypocotyl sections. The xylem area is outlined in red, the phloem area in green, whilst examples of enlarged cells filled with *P. brassicae* resting spores are indicated with yellow asterisks. Panel (d) illustrates cytokinin content in rosettes and root systems of Col‐4 and *Atabcg14* plants.

We followed our cytokinin studies with the analysis of disease progression in the model plant Arabidopsis using *Atabcg14* mutant, displaying defects in the loading of phloem‐synthesised cytokinins to xylem and their subsequent acropetal transport as well as compromised phloem unloading at the target aerial organs (Figure 6) (Ko et al., 2014; Zhang et al., 2014b). Overall, the *abcg14* mutant plants were smaller than their corresponding wild‐type (Col‐4) genotype (Figure 6a). We found that despite visible differences in morphology (Figure 6a) and significantly smaller diameters of clubroot‐induced galls (Figure 6b), disease progression in the *Atabcg14* mutant remained unaffected (Figure 6c). *P. brassicae* developed resting spores in both *Atabcg14* mutant and Col‐4 control plants (Figure 6c). We then measured cytokinins in rosettes and belowground parts of Col‐4 and *abcg14* mutants to check cytokinin accumulation in these plants during early stages of clubroot disease. Our measurements have shown strong accumulation of cytokinins in the belowground parts of the *abcg14* mutants and differential patterns of the host reaction (Col‐4 vs. *abcg14*) to *P. brassicae* infection (Figure 6d). At 12 DPI, Col‐4 controls responded to infection with decreases in cytokinins in the underground parts and increases in the aboveground parts, whereas in *abcg14* we observed opposite patterns. As previously reported, the *abcg14* mutant accumulated higher levels of active (tZ and cZ) forms in underground organs than Col‐4 wild‐type controls (Table 2; Figure S6). Mutant plants also accumulated higher levels of riboside‐type cytokinins (CKRs) in their root systems and low levels of these components in the aboveground parts. In addition to that, the belowground parts of *abcg14* mutants had higher levels of glycosylated, reversibly inactivated, forms (Table 2; Figure S6). Altogether, the observed patterns suggest that *abcg14* plants accumulate high levels of cytokinins at the site of synthesis and face severe problems with redistribution of these hormones (also in the aboveground part). This severe impact on cytokinin transport was further reflected in the plant response to *P. brassicae*. Wild‐type Col‐4 plants quickly (already at 7 DPI) responded to the infection with decreases in the active (tZ) form of cytokinin and such an effect was not observed in the mutant (Figure S6). At 12 DPI, *P. brassicae*‐inoculated Col‐4 underground parts showed decreases in both active (tZ) and transport or signalling involved (tZR) cytokinin forms, and this was not observed in the *abcg14* mutant (Figure S6).

**Table 2:** Changes in cytokinin content in Atabcg14 and Col‐4 plants observed upon *Plasmodiophora brassicae* infection

	7 DPI				12 DPI			
	Col‐4 NON	Col‐4 INF	*abcg14* NON	*abcg14* INF	Col‐4 NON	Col‐4 INF	*abcg14* NON	*abcg14* INF
Hypocotyls								
tZ	14.82 ± 5.32^a^	8.97 ± 0.86^a^	13.98 ± 1.72^a^	17.53 ± 4.94^a^	22.83 ± 4.32^b^	11.41 ± 1.8^a^	10.71 ± 2.13^a^	14.28 ± 1.22^a^
iP	7.07 ± 1.35^a^	9.97 ± 0.85^a^	11.78 ± 0.45^a^	20.69 ± 4.78^b^	11.91 ± 0.99^a^	10.93 ± 1.00^a^	19.39 ± 1.34c	15.07 ± 0.76^b^
cZ	24.57 ± 1.57^a^	17.30 ± 3.64^ab^	13.56 ± 1.86^a^	11.75 ± 1.88^a^	5.90 ± 1.26^a^	5.09 ± 0.35^a^	3.23 ± 0.59^a^	5.86 ± 3.93^a^
tZR	118.42 ± 13.65^a^	98.67 ± 6.83^a^	321.32 ± 51.73^b^	374.46 ± 112.37^b^	341.08 ± 91.67^ab^	193.57 ± 16.92^a^	356.18 ± 67.32^b^	419.08 ± 47.38^b^
iPR	65.92 ± 6.06^a^	93.81 ± 14.16^a^	171.72 ± 23.87^a^	252.02 ± 128.32^a^	138.63 ± 34.71^a^	120.08 ± 6.41^a^	333.05 ± 67.82^b^	271.93 ± 41.39^b^
cZR	144.24 ± 28.29^b^	73.68 ± 11.83^a^	61.18 ± 14.38^a^	46.00 ± 10.55^a^	27.99 ± 9.19^b^	17.00 ± 4.25^ab^	7.82 ± 2.97^a^	10.89 ± 1.10^a^
DHZR	1.60 ± 0.06^a^	1.64 ± 0.04^a^	15.99 ± 1.69^b^	12.00 ± 1.80^b^	1.38 ± 0.24^a^	1.78 ± 0.04^a^	6.88 ± 3.26^b^	13.02 ± 1.55^c^
tZ9G	171.66 ± 13.39^a^	130.26 ± 8.96^a^	369.76 ± 34.28^b^	399.42 ± 60.02^b^	210.18 ± 26.95^ab^	106.86 ± 5.58^a^	204.10 ± 63.84^b^	257.51 ± 22.32^b^
iP9G	9.53 ± 0.37^ab^	13.10 ± 1.10^b^	7.72 ± 1.02^a^	8.38 ± 2.02^a^	10.34 ± 1.32^b^	14.06 ± 0.45^c^	4.46 ± 1.31^a^	6.50 ± 0.49^a^
cZ9G	1.85 ± 0.27^a^	1.86 ± 0.15^a^	9.27 ± 1.34^b^	10.73 ± 2.19^b^	1.79 ± 0.36^a^	2.65 ± 0.81^ab^	5.96 ± 2.54^bc^	9.64 ± 0.91^c^
DHZ9G	0.82 ± 0.06^a^	0.65 ± 0.04^a^	15.57 ± 1.69^b^	13.04 ± 1.80^b^	0.74 ± 0.24^a^	0.39 ± 0.04^a^	7.30 ± 3.26^b^	10.24 ± 1.55^b^
tZMP	0.00 ± 0.00^a^	0.00 ± 0.00^a^	27.36 ± 2.58^c^	18.47 ± 5.15^b^	25.17 ± 12.17^ab^	13.46 ± 3.19^a^	95.17 ± 52.87^b^	43.61 ± 21.43^ab^
iPMP	0.00 ± 0.00^a^	0.00 ± 0.00^a^	17.62 ± 3.63^ab^	23.30 ± 12.67^b^	28.02 ± 7.15^a^	37.51 ± 7.51^a^	86.46 ± 21.88^b^	86.35 ± 11.91^b^
cZMP	27.99 ± 1.13^b^	23.24 ± 4.01^ab^	20.01 ± 0.52^a^	13.37 ± 5.55^a^	23.90 ± 3.02^b^	17.89 ± 1.85^ab^	15.49 ± 1.48^a^	17.09 ± 2.77^a^
Leaves								
tZ	11.12 ± 1.53^c^	5.98 ± 0.95^b^	0.00 ± 0.00^a^	0.00 ± 0.00^a^	0.00 ± 0.00	0.00 ± 0.00	0.00 ± 0.00	0.00 ± 0.00
iP	2.94 ± 0.53^a^	2.44 ± 0.25^a^	3.16 ± 0.29^a^	4.19 ± 0.18^b^	2.85 ± 0.14^a^	3.37 ± 0.34^ab^	4.53 ± 1.11^b^	3.06 ± 0.50^ab^
cZ	5.05 ± 1.14^a^	5.68 ± 0.57^ab^	7.30 ± 0.69^b^	7.71 ± 0.85^b^	4.36 ± 0.47^a^	3.80 ± 0.52^a^	6.80 ± 1.13^b^	8.02 ± 1.12^b^
tZR	32.64 ± 8.27^b^	9.85 ± 0.25^a^	3.46 ± 0.55^a^	4.13 ± 1.2^a^	16.85 ± 0.40^c^	19.81 ± 0.81^d^	4.86 ± 0.54^b^	2.68 ± 0.28^a^
iPR	6.43 ± 2.86^b^	1.47 ± 0.20^a^	2.83 ± 0.87^ab^	4.67 ± 1.26^ab^	3.47 ± 0.10^ab^	4.39 ± 0.27^b^	3.68 ± 0.38^a^	2.72 ± 0.68^ab^
cZR	7.43 ± 1.72^b^	3.70 ± 0.44^a^	3.00 ± 0.73^a^	3.58 ± 0.37^a^	4.92 ± 0.04^b^	2.62 ± 0.76^a^	3.37 ± 0.14^a^	5.05 ± 0.26^b^
DHZR	0.00 ± 0.00	0.00 ± 0.00	0.00 ± 0.00	0.00 ± 0.00	0.00 ± 0.00	0.00 ± 0.00	0.00 ± 0.00	0.00 ± 0.00
tZ9G	285.74 ± 38.85^b^	267.02 ± 15.99^b^	23.37 ± 1.60^a^	26.30 ± 2.46^a^	192.84 ± 7.13^a^	197.55 ± 27.3^a^	27.02 ± 1.82^b^	25.34 ± 4.69^b^
iP9G	36.60 ± 1.01^ab^	33.67 ± 2.50^a^	43.58 ± 0.56^ab^	36.43 ± 6.53^b^	28.72 ± 1.05^a^	28.72 ± 1.10^a^	35.02 ± 3.42^b^	33.15 ± 0.86^ab^
cZ9G	10.17 ± 1.14^b^	10.51 ± 0.23^b^	1.32 ± 0.17^a^	1.69 ± 0.04^a^	13.90 ± 2.47^b^	18.95 ± 0.95^c^	1.63 ± 0.15^a^	1.61 ± 0.11^a^
DHZ9G	0.52 ± 0.05^b^	0.49 ± 0.15^b^	0.00 ± 0.00^a^	0.00 ± 0.00^a^	0.70 ± 0.09^b^	0.92 ± 0.08^c^	0.00 ± 0.00^a^	0.00 ± 0.00^a^
tZMP	1667.88 ± 104.57^c^	1025.46 ± 252.73^b^	296.85 ± 37.36^a^	186.69 ± 90.42^a^	772.92 ± 232.43^c^	1285.56 ± 77.42^b^	254.71 ± 28.59^a^	128.79 ± 32.51^a^
iPMP	296.47 ± 117.19^a^	166.92 ± 13.72^a^	276.65 ± 8.40^a^	264.02 ± 101.44^a^	366.50 ± 17.32^a^	500.25 ± 19.43^a^	525.18 ± 127.22^a^	363.98 ± 45.43^a^
cZMP	183.82 ± 24.63^b^	153.34 ± 13.53^a^	117.33 ± 14.58^a^	127.02 ± 14.38^a^	155.82 ± 12.86^b^	121.43 ± 11.74^ab^	118.12 ± 16.91^a^	132.32 ± 14.29^ab^

The mean value of 3 individual biological samples is provided along with SD. Statistical differences calculated for 3 biological repeats using one‐way ANOVA followed by Tukey's test are provided in a superscript. Different letters indicate significant differences between means for the particular cytokinin form.

Based on our results obtained from studies on Arabidopsis, we can say that tight regulation of cytokinin distribution across the host is essential for *P. brassicae*‐stimulated cambial proliferation, however, this does not necessarily affect the disease progression. In previous work where we used *ipt1;3;5;7* cytokinin biosynthesis mutant, a significant delay in disease progression was observed (Malinowski et al., 2016). Here, we see that disruption of cytokinin redistribution decreases gall size and overall morphology of the infected plant without exerting a delaying effect on the *P. brassicae* life cycle and disease progression (Figure 6a–d). In light of these results, it seems that the local availability and biosynthesis of cytokinins support *P. brassicae* development, whereas cytokinin redirection, most likely, integrates nutrient signalling with cellular responses to modulate host organ growth in *P. brassicae*‐infected plants.

## DISCUSSION

### Local perception of *P. brassicae* invasion in underground organs triggers phloem‐mediated long‐distance systemic signalling

**Figure 7:**
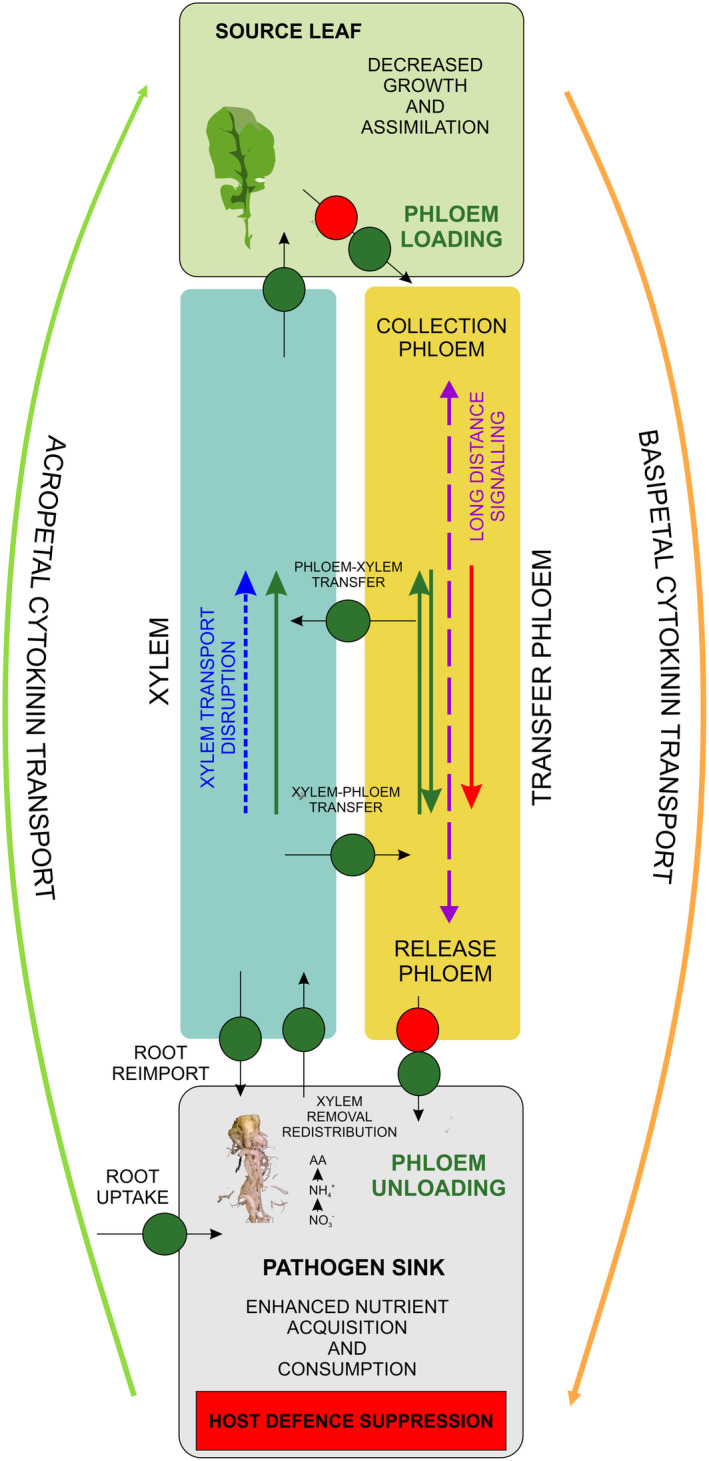
Overview on the possible routes of long‐distance coordination and repartitioning that occurs in *Plasmodiophora brassicae* infected OSR plants. Infected hosts decrease its photosynthetic activity and aboveground organ growth. This results in a lower supply of carbon‐ and nitrogen‐rich components. Despite the overall deficits of nutrients and stunted growth the host transport machinery is dominated by the strong pathogen sink in developing galls. This can be manifested as differential expression of amino‐acid and nitrate transporters as well as carbohydrate transporters having possible roles in loading at the source and unloading at the sink. Transcriptomic changes observed in dissected phloem cells also show that observed host adaptive growth and defence responses are accompanied by long‐distance signalling (purple arrow) and cytokinins may play an important role in this process. It is known that CRFs may be involved in both nitrogen status and defence response mediation. This signalling, and precise regulation of the circular long‐distance distribution of the iPR/iP type cytokinins, as well as their loading and unloading, shapes plant growth responses and source/sink relations like photoassimilate accumulation at the pathogen site, or host defence reactions like the movement of nutrients away from the pathogen site, as suggested by McIntyre et al. (2021). Possible routes of carbohydrate transport are labelled in red whereas nitrogen is marked in green. Dark blue arrow indicates disrupted water transport in impacted xylem tissue of the clubroot‐infected host. The AtABCG14‐mediated circular long‐distance transport of iP‐type CKs is pictured by the orange (basipetal) and bright green (acropetal) arrows. This aspect were reviewed in Zhao et al. (2023). Colonising pathogen gradually suppresses defence in the underground part of the host, whilst in aboveground numerous defence responses, possibly decreasing host assimilation and modulating organ growth, are observed.

Infection and further colonisation of plants by *P. brassicae* take place in the belowground parts of hosts. For this reason, long‐distance signals are required to deliver necessary information that would allow the host to launch adaptive responses in the upper parts. In the case of *P. brassicae* cortical invasion, developing plasmodia may have the ability to digest cell walls, enter the inner part of the cell and acquire nutrients *via* phagocytosis of subcellular content (Donald et al., 2008; Garvetto et al., 2023). One can imagine that the host reaction to such invasive and damage‐inflicting microorganisms will share some similarities with wound‐triggered responses. In such a situation, presumably the sequence of events leading to systemic response starts with rapid generation of electric signals, calcium fluxes and respiratory burst for transmitting information from root to shoot leading to JA synthesis in the upper part (Toyota et al., 2018). This phenomenon was described in the case of root colonisation by the root knot nematode *Meloidogyne incognita—*an interaction that shares numerous similarities with clubroot infection (Wang et al., 2019). Due to the fact that *P. brassicae* is a soil‐borne pathogen and the infection step requires secondary infection it is quite difficult to monitor rapid responses; nevertheless, the enrichment in GO terms representing reactive oxygen species generation has been previously observed at 7 and 14 DPI in root systems of susceptible *B. napus* variety ‘Laurentian’ whereas in resistant cultivar ‘Brutor’ this response was prominent only at 7 DPI (Galindo‐González et al., 2020). In our dataset, we also observe these responses in laser‐dissected phloem cells (Figure S2a–d), however, there are differences between patterns observed in phloem‐derived from hypocotyls (where the pathogen sets up its niche) and petioles of fully expanded leaves (non‐colonised upper part). In phloem from belowground parts, we see a striking difference between 7 and 12 DPI. The early time points are dominated by increases in the JA and GLN DEGs, whereas at 12 DPI remarkable decreases in the JA and aliphatic glucosinolate‐related transcripts are seen. At the same time, indolic glucosinolate‐related transcripts increase at 12 DPI. This may reflect to some extent how the pathogen modifies local responses to shape its own physiological sink in a susceptible host. It has been shown previously that indolic glucosinolates may act as the building blocks for auxin synthesis during gall formation (Ludwig‐Müller, 2009). In the phloem dissected from petiole, this differential pattern between 7 and 12 DPI was not observed and JA and GLN responses were maintained also in the later time point. This points to the importance of phloem‐mediated long‐distance coordination of further susceptible host adjustments, for example, the possible role of JA signals in modulation of plant organ growth. The occurrence of such long‐distance mediation is further supported by the fact that in clubroot‐infected *B. napus* phloem exudates collected at 12 DPI, we were able to find elevated levels of glutamate (Figure 3c). It is already known that this component, alongside electrical signals and Ca^2+^ fluxes is involved in systemic transduction of defence signals (Shao et al., 2020; Suda & Toyota, 2022; Toyota et al., 2018). We also observed elevated levels of GABA and hArg during the early stages of clubroot disease progression in *B. napus* phloem exudates (Figure 3c). It is known that plants modify nitrogen metabolism to synthesise and transport defence compounds such as GABA and hArg. For instance, GABA has been well described as one of the primary metabolites rapidly accumulated in plants under different stresses, mainly as an antioxidative response and as an alternative pathway to obtain carbon skeletons for keeping the metabolic activity *via* the GABA shunt when the stress conditions compromise photosynthesis and, hence, photoassimilate production (Podlešáková et al., 2019). Furthermore, hArg has been described as the main precursor of guanidine, a nitrogen‐rich compound whose function in plants is unclear but has been associated with nutrient alteration and defence mechanisms (Funck et al., 2023). This, together with the visible presence of the pathogen in the hypocotyls of *B. napus* at 12 DPI (Figure 1c), can be linked to the need of the host to adjust its growth and physiology to cope with disease progression. At 12 DPI in infected hypocotyl‐derived phloem cells, we can also observe enrichment in SA‐related transcripts. Salicylic acid is also involved in systemic responses, however in contrast to JA it does not require long‐distance transport to establish systemic acquired resistance (SAR) (Parker, 2009; Truman et al., 2007). Increased SA levels and enrichment in SA transcriptional signatures were previously observed in Arabidopsis plants exhibiting tolerance towards clubroot (Jubault et al., 2013; Lemarié et al., 2015) and resistant cultivars of *B. napus* (Galindo‐González et al., 2020; Jayasinghege et al., 2023). In a compatible reaction, where the host is susceptible to the pathogen, triggered SA responses cannot lead to effective SAR and must be suppressed so the pathogen can bypass the host defence. Together, transcriptomic changes observed in phloem reflect a delicate balance in defence responses present in susceptible OSR plants and depict the difference between colonised belowground parts of a plant and non‐colonised aboveground organs (Figure 7).

### Expression patterns observed in laser dissected phloem cells as well as sugar and amino‐acid content of phloem sap in *P. brassicae*‐infected *B. napus* plants indicates that this tissue coordinates host responses to reduced nutrient supply during clubroot disease progression

Due to its role in the transport of assimilates and redistribution of nitrogen, the phloem acts as the main conduit involved in the long‐distance adjustment of energy consumption within the plant. Upon clubroot infection followed by the pathogen sink formation and consumption of host nutrients, the plant will face a sudden nitrogen and carbon deficit. Disease development quickly leads to a decrease in photosynthesis and transpiration (Figure S5b,d). This is further reflected by a reduction in stomatal conductance and intercellular CO_2_ concentration (Figure S5a,c). Based on the maximum photochemical efficacy (*F*_v_/*F*_m_) parameter that did not differ significantly between mock‐inoculated and clubroot‐infected *B. napus* plants during early steps of disease progression (12 DPI), we can assume that observed differences are mainly related to the change in the trophic relations and further plant organ growth decreases than photosystem damage (Figure S5e). Knowing the fact that the pathogen needs to establish its own physiological sink, increased consumption of the host nutrients is not surprising. During clubroot infection the developing gall becomes the dominating sink, therefore the majority of transport is redirected towards it. Accordingly, the upregulation of SWEET sucrose transporters was observed in the phloem of developing galls (Walerowski et al., 2018) since, due to the accumulation of carbohydrates (Evans & Scholes, 1995), sucrose has to be actively unloaded to the higher concentration niche. In phloem exudates collected from petioles of fully expanded leaves of *B. napus* we found a substantial drop in phloem sap amino acid and sucrose content (Figures 3 and 4, respectively), which further reflects a decrease in the infected host's photoassimilation. Despite the dominance of the pathogen sink, the host adapts to the unfavourable situation by decreasing organ growth since it cannot supply its own organs with sufficient amounts of nutrients. In the case of some other stresses, such as drought, where plants respond with changes in growth allocation (e.g., root growth activation) or increasing phloem sap osmotic potential to avoid transport discontinuity, the amino acid content in phloem increases (Blicharz et al., 2021), whereas in clubroot‐infected plants AA and sucrose levels decreases. Our transcriptomic data obtained from laser‐dissected phloem shows that this is accompanied by differential expression of factors whose activity may be involved in nitrogen redistribution (*NRT/NPF*, *AAP*, *LHT* and *UMAMIT* transporters). At present, we cannot provide functional evidence on the role of particular gene products, however, observed patterns clearly indicate an intense reshuffling of nitrogen‐rich components. It seems clear that nitrogen and carbon repartitioning will play an important role in systemic signal transduction that shapes the whole biotrophic interaction. *P. brassicae* has limited capacity for direct modulation of nitrogen uptake and metabolism (Schwelm et al., 2015). This does not change the fact that it creates a strong physiological sink that facilitates high nitrogen and carbohydrate consumption. Disease leads to growth suppression and the host needs to reshuffle nutrients to maintain the most critical functions or protect particular aspects related to its physiology. The recent work of Aigu et al. (2022) points out that the genetic ability to regulate nitrate repartitioning or assimilation may result in a certain degree of host tolerance to *P. brassicae* infection. Here, in a susceptible *B. napus* genotype, we show that amino acid content in phloem sap collected from petioles of fully expanded leaves generally decreases and the main group of increasing components are Pro, Gln and GABA, hArg and Ile. The action of these components was previously linked to the systemic root‐to‐shoot wound signalling (Hönig et al., 2023; Shao et al., 2020) and in fact these responses were also observed at the transcriptional level in the phloem of *P. brassicae* infected *B. napus* plants. There are also reports showing that under low N availability levels of Pro, Glu and GABA increase in phloem (Chardon et al., 2022; Perchlik & Tegeder, 2017). Results of our transcriptome profiling and targeted metabolite measurements suggest the role of observed changes in the long‐distance‐mediated signalling and are in agreement with previously postulated multiple roles of nitrogen in shaping plant responses (O'Brien et al., 2016). Here with the help of laser dissection microscopy, we were able to show that the N and C regime in the host is regulated at the system level and that the phloem acts as a conduit coordinating this mechanism (Figure 7). We cannot exclude also the situation when host stimulates redirection of nutrients to its own sink to restrict its availability to the pathogen sink, therefore such scenario should also be functionally tested in the future. Biotrophy can be very dynamic so the responses at the nitrate transporter or amino acid transporter level may reflect both defence and adaptive developmental reactions in the host.

### Vascular tissue‐mediated cytokinin transport shapes growth trade‐offs in *P. brassicae* infected plants but does not play a direct role in the pathogen life cycle progression

Due to the fact that phloem mediates the redirection of nutrients between source and sink, there is a high possibility that it is a central hub coupling signalling with nutritional status. The results of our phloem transcriptome profiling show that host responses include nitrogen repartitioning, which is probably related to an increased demand, and later on usage, of nitrogen by the pathogen. This subject was recently linked to the degree of tolerance to clubroot in *B. napus* (Aigu et al., 2022). The work of Abualia et al. (2022) shows that in Arabidopsis nitrate status in roots is further translated to growth responses with the help of cytokinins that upon acropetal transport modulate the expression of *CRFs* in shoots and this has a further impact on auxin PIN‐mediated transport regulation. Typically, in healthy plants the uptake of nitrates from soil induces phloem‐specific expression of the *IPT3* gene, whose product is involved in cytokinin biosynthesis. Synthesised cytokinins are then unloaded from the phloem to the xylem with the help of the high‐affinity cytokinin transporter ABCG14, which is also involved in the further unloading of cytokinins *via* phloem in the upper parts. We found elevated levels of the iP‐type cytokinins in phloem sap collected from fully expanded leaves of the *P. brassicae*‐infected *B. napus* at the proliferative stage of gall formation. We found that both active (iP) and transport (iPR) forms were elevated. It is known that CKRs can act systemically and are able to trigger cytokinin downstream responses (Nguyen et al., 2021). Elevated levels of iPR observed in *B. napus* in phloem upon *P. brassicae* infection could be amongst the factors responsible for observed changes in *CRF*s expression in phloem. Previous works on Arabidopsis suggest that the phloem‐mediated basipetal transport of the iP‐type cytokinins may be involved in orchestrating the vascular patterning in roots (Bishopp et al., 2011). The importance of the iP‐type cytokinins for pathogen‐feeding site formation (cellular proliferation) was previously shown in root‐knot nematode interactions with Arabidopsis (Siddique et al., 2015). On the other hand, Sasaki et al. (2014) have shown that shoot‐derived cytokinins negatively regulate nodulation in *Rhizobia‐*colonised *Lotus japonicus* plants. At present, we are not sure about the exact role of the phloem‐transported iP‐type cytokinins during clubroot. We found that disrupted cytokinin transport in the *abcg14* Arabidopsis mutant did not impact the pathogen development (Figure 6). So far only the cytokinin biosynthesis disruption has been shown to affect *P. brassicae* life‐cycle progression (Malinowski et al., 2016) and attempts to impair cytokinin signalling did not bring unequivocal results (Bíbová et al., 2023). Here we show that in Arabidopsis, disrupting cytokinin transport using *abcg14* leads to reduced size of galls, which is in agreement with the previous findings on the contribution of xylem and phloem transport to the cambial activation (Matsumoto‐Kitano et al., 2008; Sakakibara, 2021). Yang et al. (2021) have shown that cytokinin‐stimulated cambial proliferation in roots is mediated by the DREAM complex factors. This stays in agreement with our previous work where we identified the MYB3R4 involvement in clubroot‐driven cambial proliferation and gall development in *P. brassicae*‐infected Arabidopsis plants (Olszak et al., 2019). From our results, it appears that local cytokinin synthesis in developing galls is responsible for the pathogen sink maintenance whereas other aspects are more likely associated with systemic shaping of the growth trade‐offs related to nutrient availability at the level of signalling or cell proliferation in the infected host plant (Figure 7). Apparently, *P. brassicae* infection leads to systemic reprogramming of cytokinin responses in phloem tissue (Figure 7), however, the amplitude of these responses can fluctuate during disease, thus, more detailed work should be performed to dissect this phenomenon. The use of Arabidopsis with its vast availability of tools seems to be the most plausible direction. Another critical aspect that should be taken into account is the interplay between the actual nitrogen form availability and the rate of cytokinin transport in xylem and phloem in clubroot‐infected plants. To the best of our knowledge, such experiments were performed only for healthy *B. napus* plants (Heuermann et al., 2021) and this may be a promising direction to work on in the near future.

## MATERIALS AND METHODS

### Plant growth conditions

Experiments were performed on the *B. napus* winter oilseed rape cultivar ‘Harry’ (Syngenta, UK). Plants were grown in Klasmann Select LT0‐11 substrate supplied weekly with 0.25 g L^−1^ of Peters Professional Winter Grow Special 20‐10‐20 NPK + TE (Trace Elements—micronutrients). The amount of water and fertiliser was adjusted to keep the optimal N content advised for this crop at the rosette stage (30 kg ha^−1^). Plants were grown in a fully automated greenhouse at 250 μmol m^−2^ sec^−1^ light intensity and 16/8 h day/night photoperiod and 22°C day/20°C night temperature regime. Each 6‐day‐old plant was inoculated by pipetting 4 mL of a 5 × 10^6^ spores mL^−1^ suspension of *P. brassicae* pathotype P1+ (Ochoa et al., 2023; Zamani‐Noor et al., 2022) in the soil next to the hypocotyl whilst control plants were mock‐inoculated with 4 mL of water.

In experiments performed on Arabidopsis, *abcg14* mutant (Ko et al., 2014) the appropriate wild‐type accession Columbia‐4 (Col‐4) was used as a control. Plants were grown and inoculated according to Malinowski et al. (2012) and the P1+ *P. brassicae* pathotype was used.

To block phloem transport previously described (Vatén et al., 2011) Arabidopsis *APL::XVE‐cals3m* estradiol‐inducible line was used. In this system, the estradiol induction of the gain of function *cals3m* gene expression leads to callose deposition in phloem SE cells. In our experiment growth, *P. brassicae* inoculation and plant collection were carried out the same way as for other experiments with Arabidopsis described above. The additional factor was the induction of the *cals3m* gene expression. Estradiol treatment started when plants were already growing in pots, 3 days before inoculation with *P. brassicae* or control mock inoculation. For the induction (+EST) 0.5 mL of a 20 μM β‐estradiol (Sigma–Aldrich, Merck KGaA, Darmstadt, Germany) was applied by pipetting in the soil, next to the hypocotyl. For non‐induced combination (−EST) plants 0.5 mL of water was applied in the same manner. Treatments were repeated every second day, until the collection time (26 DPI).

### Tissue fixation and embedding of samples for LCM analysis

For the transcriptomic experiment hypocotyls or fragments of petioles from the fully expanded first true leaf of the *P. brassicae* or mock‐inoculated *B. napus* plants were collected at 7 and 12 DPI, respectively. Samples were immediately fixed with ice‐cold fixative solution (5:1 ethanol:acetic acid [v/v]), vacuum‐infiltrated twice for 5 min and incubated at 4°C for 3 h. The solution was subsequently replaced with fresh ice‐cold fixative (5:1 ethanol:acetic acid [v/v]) and incubated at 4°C for at least 12 h. Fixed tissue was dehydrated at 4°C in a graded series of ice‐cold ethanol (1 h each of 85, 95, 100, 100 and 100% overnight). Samples were infiltrated with an increasing dilution series of Histo‐Clear II:ethanol (1 h each of 1:3, 1:1, 3:1 [v/v]), followed by incubation in 100% Histo‐Clear II three times for 30 min at room temperature. Samples were then infiltrated with 1:1 (v/v) Histo‐Clear II:Paraplast X‐tra solution for 1 h at 55°C, followed by incubation in 100% Paraplast X‐tra for 2 h at 55°C. The solution was replaced with fresh 100% Paraplast X‐tra, incubated overnight at 55°C, replaced twice with fresh 100% Paraplast X‐tra and incubated each time for 2 h at 55°C. Tissue samples were then embedded in 100% Paraplast X‐tra and left to solidify for 2 h.

### Laser‐capture microdissection

Transverse sections (10 μm) of the embedded material were cut using a Leica RM 2135 rotary microtome, stretched in a DEPC‐treated water bath, transferred onto RNase‐free Arcturus^®^PEN Membrane Glass Slides (Applied Biosystems) and dried on a hot plate at 42°C. Slides were deparaffinised by incubation in Histo‐Clear II 5 times for 5 min, washed twice in 100% ethanol for 5 min, left to evaporate at room temperature and stored at −70°C until LCM. Prior to microdissection cross‐sections on the slides were stained with toluidine blue‐saturated ethanol and dried at 37°C. Phloem tissue was dissected using a Leica LMD7 (Leica Microsystems CMS GmbH, Wetzlar, Germany) microscope and captured into the caps of 200 μL low‐binding tubes. Laser dissection parameters were as follows: power 25–30, aperture 30–35, speed 40, specimen balance 15 and pulse frequency 228. Sample collection was verified by checking the contents of collector caps under the microscope. Each biological replicate contained 2000–10 000 phloem regions (corresponding to 17–45 mm^2^ of a tissue area) dissected from 3 slides (each slide corresponding to a different specimen) and collected in one tube cap. Four biological replicates were collected for each experimental combination. Unfortunately, during further processing some RNA samples did not pass the quality control, therefore we ended up with 3–4 biological replicates per condition, that were used as a starting material for the library preparation.

### RNA extraction, library preparation and transcriptome sequencing and analysis

Immediately after dissection, 10 μL of extraction buffer from the Arcturus™ PicoPure™ RNA Isolation Kit (Applied Biosystems by Thermo Fisher Scientific Vilnus, Lithuania) was added to each sample cap. The material was collected at the bottom of the tube by brief centrifugation at 800*g* and the samples were incubated at 42°C for 30 min. Subsequent RNA extraction, including the on‐column DNase treatment, was performed according to the manufacturer's protocol. RNA quality and quantity were assessed using the Agilent Bioanalyzer 2100 and RNA 6000 Pico Kit. Verification of methodology for phloem specificity of the dissected material was performed using qRT‐PCR with *PP2A‐1* (*A09p17880.1_BnaDAR*), *SUC2* (*A09p42030.1_BnaDAR*) and *APL* (*A07p44500.1_BnaDAR*) as positive control genes (Table S2). First‐strand cDNA synthesis was performed on 150 ng of RNA with the RevertAid H Minus First Strand cDNA Synthesis Kit (Thermo Fisher Scientific) following manufacturer's instructions. The RT‐qPCR reactions were performed using the LightCycler 480 instrument (Roche) and the SensiMix SYBR No‐ROX Kit (Bioline). Final cDNA template concentration in the reaction was 0.4 ng μL^−1^. The program was as follows: 10 min 95°C − 45 × (20 sec 95°C − 20 sec 60°C − 20 sec 72°C) ending with a melting curve generation. Relative gene expression levels were calculated and normalised with REST‐MCS software using the geometric mean of four reference genes: *UBC9* (*C08p10490.1_BnaDAR*), *UBC10* (*A10p10830.1_BnaDAR*), *ACT7* (*C02p04090.1_BnaDAR*) and *ENTH* (*A01p06100.1_BnaDAR*), (Table S1) according to the method described by Pfaffl et al. (2002). The enrichment was observed for all three tested positive control genes (Figure S7).

cDNAs for transcriptome sequencing were prepared from 5 ng of total RNA. Firstly, ribosomal RNA was removed from total RNA, followed by ethanol precipitation. After fragmentation, the first strand cDNA was synthesised using random hexamer primers. During the second strand cDNA synthesis, dUTPs were replaced with dTTPs in the reaction buffer. The directional library was ready after end repair, A‐tailing, adapter ligation, size selection, USER enzyme digestion, amplification and purification. The library was checked with Qubit and real‐time PCR for quantification and bioanalyzer for size distribution detection. Libraries were sequenced on Illumina platforms (150 base paired end sequencing; NovaSeq PE150) and for each sample 3G raw data was obtained. Both library preparation and sequencing were performed commercially by Novogene (Novogene Co. LTD, Cambridge, UK). Library reads were trimmed and filtered based on read quality and length and then mapped to the combined genome space of *B. napus* and *P. brassicae* using the Genoscope long reads assembly of the *B. napus* Darmor‐bzh cultivar (Rousseau‐Gueutin et al., 2020) and the *P. brassicae* e3 strain assembly from Genbank (GCA_900303365.2), using CLC Genomics software (Qiagen). Unfortunately, in three samples there were an unacceptable number of contaminant or artefact reads relative to those mapping to the host or pathogen genomes, hence they were excluded from subsequent analysis. For each condition 2–4 replicates were available, counts of mapped reads for each sample are detailed in Supplementary File 1. This data have been deposited in the NCBI SRA database (https://www.ncbi.nlm.nih.gov/sra), where it is available under the identifier PRJNA1139814. Counts of reads mapping to genes were analysed using the DESeq2 package in the R software environment (Love et al., 2014). Significantly differentially expressed genes were identified based on a log_2_ ratio ≥1 and a false discovery rate ≤0.05 (Storey, 2002). A library of GO‐terms for the Genoscope *B. napus* genome was generated using the OmicsBox software (BioBam) and significantly enriched terms for each DEG list were identified with a Benjamini–Hochburg adjusted *P*‐value threshold of ≤0.05. The OrthoFinder software (Emms & Kelly, 2019) was used to identify orthologous relationships between genes from *B. napus* and Arabidopsis by analysing the Genoscope Darmor‐bzh and TAIR10 proteomes in combination with other proteomes from the PLAZA4.5 dicot collection (van Bel et al., 2018). Clustering of gene expression profiles was performed with CLUSTER 3.0 (Eisen et al., 1998) and visualised using Treeview.

### Phloem exudate collection

For amino acid and sugar content measurements phloem exudates were collected from fully expended leaves of *P. brassicae‐*infected and mock‐treated *B. napus* plants at 7, 12, 16, 26 and 32 DPI using the EDTA‐assisted method described previously by Tetyuk et al. (2013). The exudation was carried out for 1 h. Exudates for cytokinin measurements were collected according to the same method at 7, 10, 14 and 26 DPI, however, the exudation time was extended to 8 h. For each experimental combination and time point five biological replicates were collected. In both experiments, each replicate contained exudates from 60 leaves (20 plants per sample and 3 leaves from each plant). Exudates were frozen in liquid nitrogen followed by lyophilisation. The fresh mass of leaves used for exudation was measured and used for calculations.

### Studies on cytokinin transport disruption in the *abcg14* mutant

For cytokinin analysis in Arabidopsis sampling was performed at 7 and 12 DPI with the aboveground and belowground parts of plants collected separately. For each combination, 3 biological repeats were collected. At the 7 DPI time point, due to the minimal amount of plant material required, each biological replicate comprised 60 plants for Col‐4 and 90 plants for the *abcg14* mutant (which is characterised by having a small root system). At the 12 DPI time point 36 plants for each biological repeat were collected.

### Extraction and determination of cytokinin, amino acid and sugar content

The determination of cytokinin (CK) content in plant material was performed as previously described by Glanz‐Idan et al. (2022). Briefly, 10 mg of lyophilised and homogenised plant material was extracted by 1.2 mL of modified Bieleski extraction solvent (Bieleski, 1964), (75/20/5; methanol/H_2_O/formic acid, MeOH/H_2_0/FA) containing a mixture of internal standards at −20°C overnight. After centrifugation (4°C, 20 000*g*, 10 min), the supernatant was collected and pellets were reextracted with an additional 0.5 mL of modified Bieleski solution at −20°C for 1 h and again centrifuged. Combined supernatants were loaded onto equilibrated 100 mg SCX 1 mL solid‐phase extraction cartridges (Agilent). Cartridges were activated by 1 mL of MeOH, equilibrated with 1 mL of 50/48/2 MeOH/H_2_O/FA, followed by a sample loading, washing by 1 mL of modified Bieleski solution and 1 mL of MeOH. Analytes were eluted by 2 mL of 4 M NH_4_OH in 60% MeOH and evaporated *in vacuo*. The sample preparation procedure for the analysis of CKs in phloem and xylem exudates required an adjustment due to the larger volumes of the buffers used for the exudate collection. Samples in a volume of up to 50 mL (buffer containing collected exudates) were adjusted with FA to a final concentration of 1 M FA, a mixture of internal standards was added and samples were loaded onto equilibrated 150 mg MCX 6 mL solid‐phase extraction cartridges (Waters) following the protocol described by Ivanov Dobrev and Kamínek (2002). Cartridges were activated using 5 mL of MeOH, followed by sample loading and washing with 5 mL 1 M FA solution and 5 mL of MeOH. CK nucleotides, bases, ribosides and glucosides were eluted as a single fraction by 5 mL 0.35 M NH_4_OH in 60% MeOH and evaporated *in vacuo*. All samples were reconstituted in 40 μL of the mobile phase. UHPLC–MS/MS analyses of plant material samples and exudate samples were performed using the NEXERA X2 modular system coupled with MS 8050 (Shimadzu, Japan) via an electrospray interface. Chromatographic separation was performed using a reversed‐phase analytical column (BEH C18, 2.1 mm × 100 mm, 1.7 μm; Waters) with corresponding pre‐column. Gradient elution was as follows: temperature 40°C, solvent A—15 mM ammonium formate pH 4, solvent B—MeOH, flow rate of 0.25 mL min^−1^ and a profile of 0.5 min 10% B, 12 min 43% B, 15 min 100% B, 16 min 100% B, 17.5 min 10% B and 21.5 min 10% B. Ion source configuration remained default as developed by the vendor. Raw data were processed using Shimadzu software LabSolutions v. 5.97 SP1.

Amino acids in plant material were determined via a recently published protocol by Ćavar Zeljković et al. (2024). For the analysis of exudates, samples were lyophilized and reconstituted into the mobile phase. UHPLC–MS/MS analyses were done on the same instrument described above. Chromatographic separation was performed on an Acquity UPLC BEH AMIDE (150 × 2.1 mm; 1.7 μm particle size) column with a corresponding pre‐column. Identification of analytes was performed in multiple reaction monitoring mode comparing the spectra with authentic standards.

The analysis of the free sugars, glucose, fructose and sucrose was performed according to the slightly modified method of O'Donoghue et al. (2004). The 25 μL of lyophilised exudates were extracted with 1 mL of deionised water and filtered. The sugars were separated on a Rezex RCM monosaccharide Ca^2+^ column (300 mm × 7.8 mm, 8 μm). The detection was performed by ELSD under a nitrogen flow of 2 L min^−1^ and a detector temperature of 80°C.

### Determination of tissue‐specific expression of amino acid and nitrate transporter gene candidates

Approximately 1.5 kb of the 5′UTR and promoter region of selected genes (Table S2) were amplified using CloneAmp HiFi PCR Premix polymerase (Takara) and subsequently fused to the EGFP coding sequence for cellular promoter activity pattern monitoring of the following genes: *A05p08480.1_BnaDAR* (*UMAMIT12* orthologue); *C08p07150.1_BnaDAR* (*UMAMIT18* orthologue); *A09p18320.1_BnaDAR* (*AAP1* orthologue); *C06p51510.1_BnaDAR* (*AAP3* orthologue) and *C07p51310.1_BnaDAR* (*NRT1.8/NPF7.2* orthologue). Constructs were prepared in Green Gate system vectors according to Lampropoulos et al. (2013) and Schürholz et al. (2018): promoter fragments were fused with 3xGFP coding sequence and UBQ10 terminator in the destination vector pGGZ003. Resulting plasmids were introduced into GV3101 (pSOUP) Agrobacterium chemically competent cells (GoldBio, St Louis MO, USA) and subsequently used for the floral dip Arabidopsis transformation according to Clough and Bent (1998). Transgenic plants were selected on 0.5 MS plates containing 10 μg mL^−1^ of hygromycin. Homozygous lines were used for observations where 1 mm transverse sections of petioles from the fully expanded leaf No. 2 of *P. brassicae* and mock‐inoculated plants were cut on an automated Vibratome Leica VT1200. Sections were counterstained with calcofluor white and observed under a Carl Zeiss AxioImager M2 automated epifluorescence microscope with Colibri LED system in multiple acquisition modes (calcofluor filter set No. 49 405/475 excitation/emission and GFP filter set No. 38 488/509 excitation emission).

### Light microscopy for anatomical change inspection

Arabidopsis *abcg14* mutant and wild‐type Col‐4 3 mm long hypocotyl fragments were collected at 26 DPI and fixed in 0.5% glutaraldehyde, 4% PFA w 1x PBS, pH 7.5 buffer. Fixed objects were subsequently dehydrated and embedded in Technovit 7100 (Kultzer). Embedded objects were sectioned on the Leica RM 2135 rotary microtome and resulting 8 μm transverse sections were stained with 0.02% toluidine blue solution. For each experimental combination sections from 5 hypocotyls (5 sections of each) were observed under transmission light on the Carl Zeiss AxioImager M2 microscope.

### Determination of photosynthetic parameters

Measurements of gas exchange and chlorophyll fluorescence were performed in mature, fully expended leaves (leaf No. 2) at 12 DPI for 10 mock and 10 *P. brassicae* inoculated OSR plants growing in separate pots. Gas exchange parameters including photosynthesis (Pn), evaporation (E), stomatal conductance (gs) and intercellular CO_2_ concentration (Ci) were recorded by LI‐6400XT Portable Photosynthesis System (Li‐Cor, Lincoln, USA). Measurements were performed under artificial light with the intensity of 150 μmol m^−2^ sec^−1^, 380 ppm CO_2_, airflow speed in the measuring chamber of 400 μmol sec^−1^, 25% humidity and 21°C temperature. To check plant photosynthetic performance at 12 DPI the HandyPEA fluorimeter (Hensatech Instruments Ltd., King's Lynn, England) was used. Maximum quantum yield parameter (*F*_v_/*F*_m_) was calculated for 20 mock and 20 *P. brassicae* inoculated OSR plants growing in separate pots. Statistically significant differences were determined by t‐test for results that showed normal distribution, or by Wilcoxon signed‐rank test for data that do not follow normal distribution with R (version 4.2.2, The R Foundation for Statistical Computing, Vienna, Austria). A *P*‐value <0.05 was considered a significant change.

## AUTHOR CONTRIBUTIONS

SB, KS and RM designed the research; SB, KS, AB‐B, DS, AK, ND, OV, SĆZ, PT and RM performed the research; SB, RM, ND, PT and WT analysed the data; RM wrote the first version of the paper and all other co‐authors were involved further preparation of the final manuscript.

## CONFLICT OF INTEREST

The authors declare that they have no known competing financial interests or personal relationships that could appear to influence the work reported in this paper.

## Supporting information


**Figure S1.** The number of genes that are differentially expressed in *Plasmodiophora brassicae*‐infected plants and overlap in phloem isolated from hypocotyl and petiole.

**Figure S2.** Enrichment of selected GO terms for differentially expressed genes. Significant DEGs were identified based on the thresholds of log_2_ ratio ≤−1 or ≥1 and a false discovery rate ≤0.05. (a–d) Visualisation of selected enriched GO term categories in the hypocotyl phloem (a) 7 DPI and (b) 12 DPI, as well as in the petiole phloem. (c) 7 DPI and (d) 12 DPI of the *Plasmodiophora brassicae‐*infected *Brassica napus* plants compared to the mock‐treated plants. Significantly enriched terms were identified with a Benjamini–Hochberg adjusted *P*‐value threshold of ≤0.05.

**Figure S3.** Effects of phloem transport disruption on *Plasmodiophora brassicae* disease severity and gall development studied in estradiol inducible *pAPL::XVE>>icals3m* line. Panel (a) shows differences in the phenotype of rosettes and hypocotyls observed in *pAPL::XVE>>icals3m* line upon *P. brassicae* infection between plants depositing callose in phloem SE cells (+EST) and plants having uncompromised phloem transport (−EST). Panel (b) shows the comparison for hypocotyl diameter. Panel (c) shows clubroot disease severity comparison at 26 DPI. Radial sections were stained with TB solution.

**Figure S4.** Promoter activity for GFP fusions of *UMAMIT 12* and *18 Brassica napus* amino acid transporters orthologues *A05p08480.1_BnaDAR* and *C08p07150.1_BnaDAR* respectively at 7 and 12 DPI in transverse sections of petioles from mock‐treated and *Plasmodiophora brassicae‐*infected Arabidopsis plants. Sections were counterstained with Calcofluor White and GFP signals from reporter genes fused to chosen promoter were observed under an epifluorescent microscope. Scale bars represent 50 μm.

**Figure S5.** Comparison of photosynthesis and gas exchange parameters in *Plasmodiophora brassicae*‐infected OSR plants versus appropriate mock‐inoculated control at 12 DPI. Panel (a) shows stomatal conductance (*n* = 10), (b) transpiration rate (*n* = 10), (c) intercellular CO_2_ concentration (*n* = 10), (d) photosynthetic efficiency (*n* = 10) and (e) maximum photochemical efficacy *F*_v_/*F*_m_ (*n* = 20). Asterisks indicate statistically significant differences at *P* ≤ 0.05 that were calculated by unpaired t‐test for results that showed a normal distribution, or by Wilcoxon signed‐rank test for data that do not follow normal distribution. [Correction added on 6 December 2024, after first online publication: the legends of Figures S4 and S5 have been swapped in this version.]

**Figure S6.** Cytokinin changes triggered by *Plasmodiophora brassicae* in Col‐4 and *abcg14* mutant. Detected components were divided into four groups based on their function in the plant—active forms (trans‐zeatin tZ, cis‐zeatin cZ and isopentenyladenine iP), inactive forms (trans zeatin glucoside tZ9G, cis zeatin glucoside cZ9G, isopentenyl adenine glucoside iP9G and di‐hydroxy zeatin glucoside DHZ9G), transport forms (trans‐zeatin riboside tZR, cis‐zeatin riboside cZR and isopentenyl riboside iPR) and primary components of cytokinin biosynthesis (trans‐zeatin monophosphate tZMP, cis‐zeatin monophosphate cZMP and isopentenyl monophosphate iPMP). Error bars represent SD values of 3 biological replicates (at 7 DPI time points each biological replicate included 60 plants for Col‐4 and 90 plants for abcg14. At 12 DPI time points 36 plants represent 1 biological repeat). Different letters indicate a significant difference between means for the particular cytokinin form according to one‐way ANOVA with Tukey's multiple comparison test.

**Figure S7.** Phloem‐specific gene enrichment test performed for dissected material. Relative expression levels of the *BnPP2A‐1*, *BnSUC2* and *BnAPL* genes were higher in dissected phloem regions in comparison to the surrounding tissue material.

**Table S1.** List of oligonucleotide primers used for the enrichment test of laser dissected material before cDNA libraries preparation and transcript sequencing.

**Table S2.** List of oligonucleotide primers used for 5′ upstream sequences cloning and preparation of constructs in a GreenGate system.

**Supplemental File 1.** Complete transcriptomic dataset for gene expression profiling in dissected phloem.

## Data Availability

The transcriptomics data has been deposited at the NCBI SRA database (https://www.ncbi.nlm.nih.gov/sra), where it is available under the identifier PRJNA1139814. Apart from that all relevant data can be found within the manuscript and its supporting materials.
