# Supplementary figures and images for: Laser dissection‐assisted phloem transcriptomics highlights the metabolic and physiological changes accompanying clubroot disease progression in oilseed rape
Source: Plant J. 2024 Nov 22;121(1):e17156. doi: 10.1111/tpj.17156 (PMC11703547; doi:10.1111/tpj.17156)

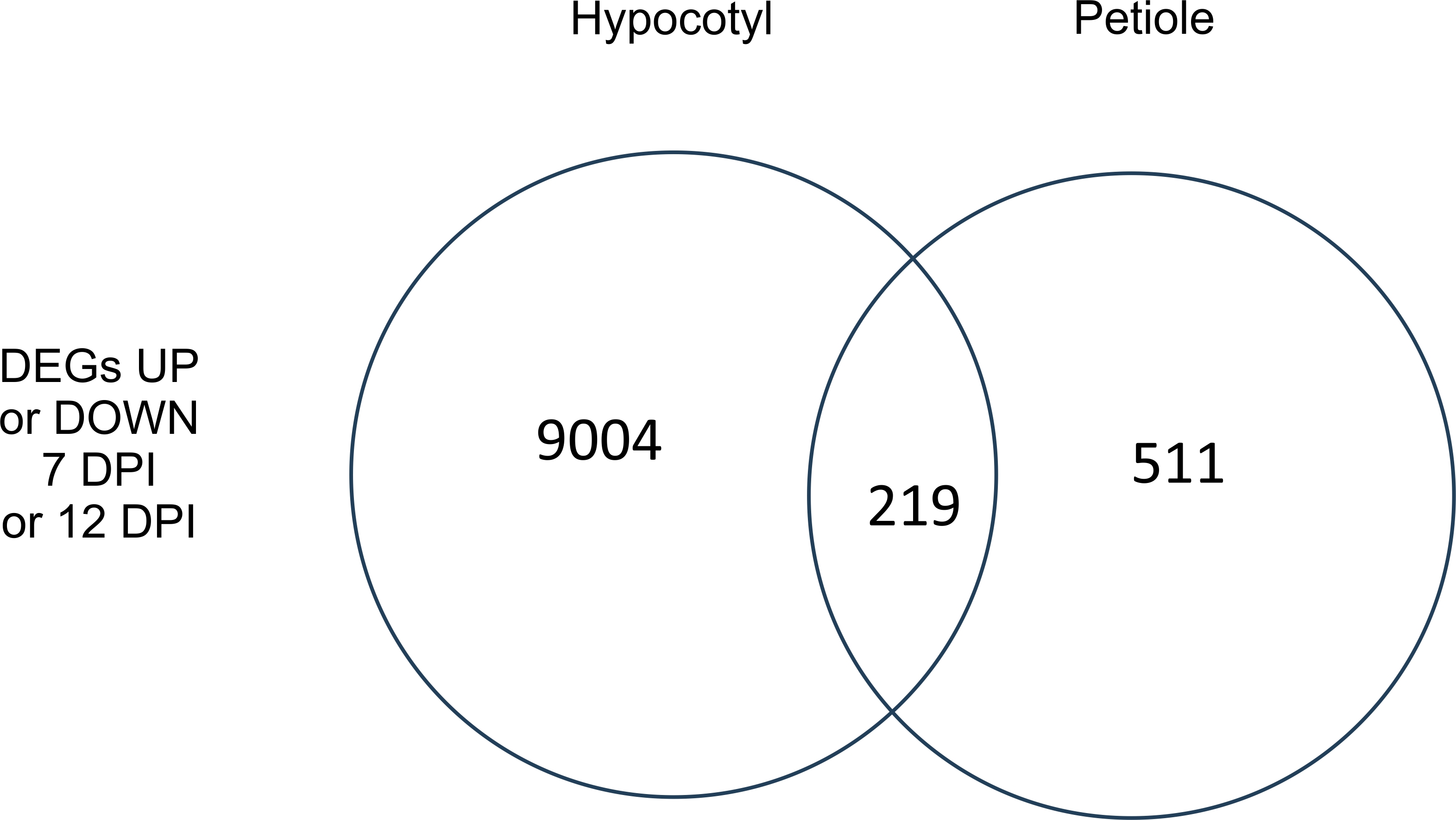

Supplement: Supplementary file 1 — Figure S1. The number of genes that are differentially expressed in Plasmodiophora brassicae‐infected plants and overlap in phloem isolated from hypocotyl and petiole. [file TPJ-121-0-s008.jpg]

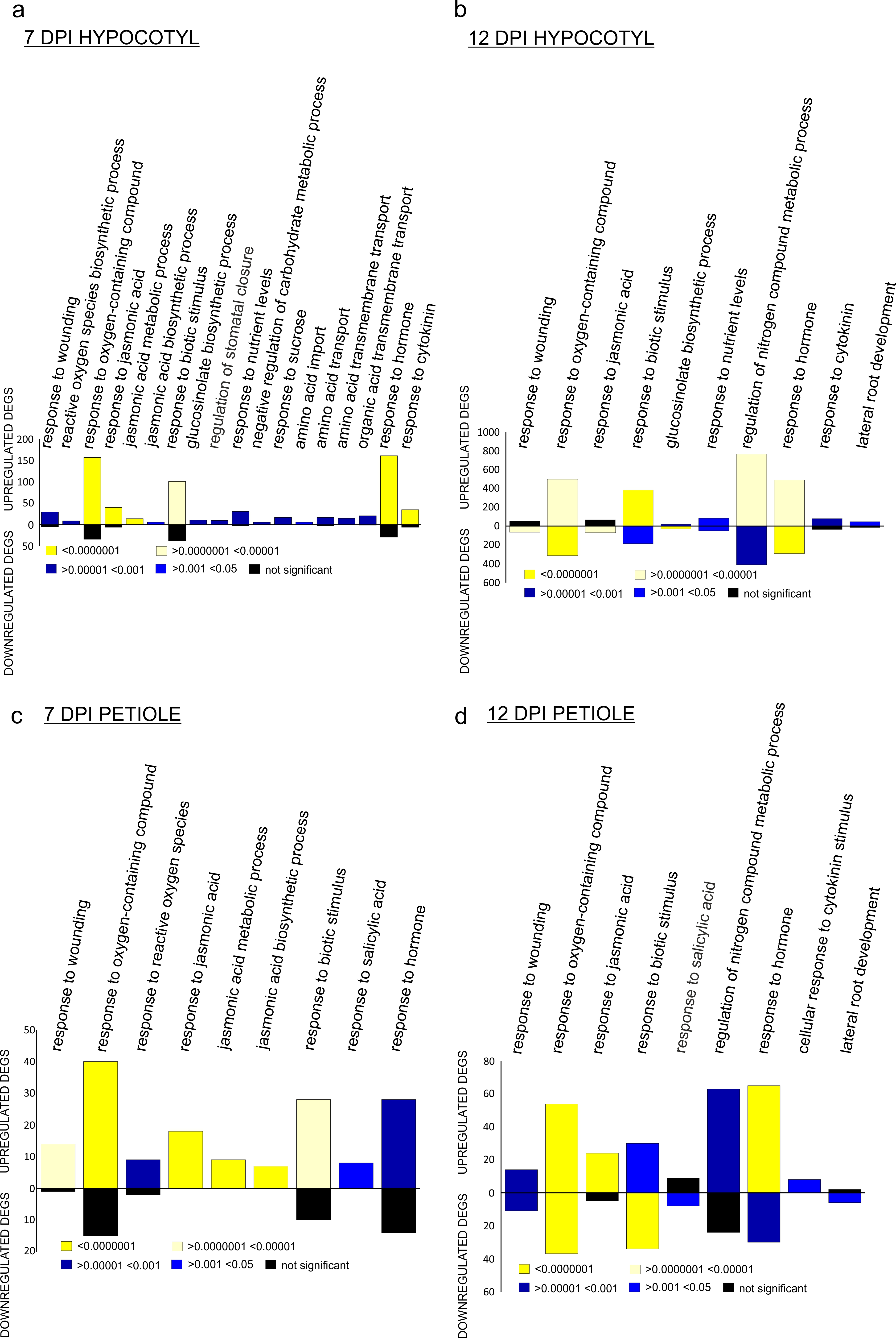

Supplement: Supplementary file 2 — Figure S2. Enrichment of selected GO terms for differentially expressed genes. Significant DEGs were identified based on the thresholds of log2 ratio ≤−1 or ≥1 and a false discovery rate ≤0.05. (a–d) Visualisation of selected enriched GO term categories in the hypocotyl phloem (a) 7 DPI and (b) 12 DPI, as well as in the petiole phloem. (c) 7 DPI and (d) 12 DPI of the Plasmodiophora brassicae‐infected Brassica napus plants compared to the mock‐treated plants. Significantly enriched terms were identified with a Benjamini–Hochberg adjusted P‐value threshold of ≤0.05. [file TPJ-121-0-s003.jpg]

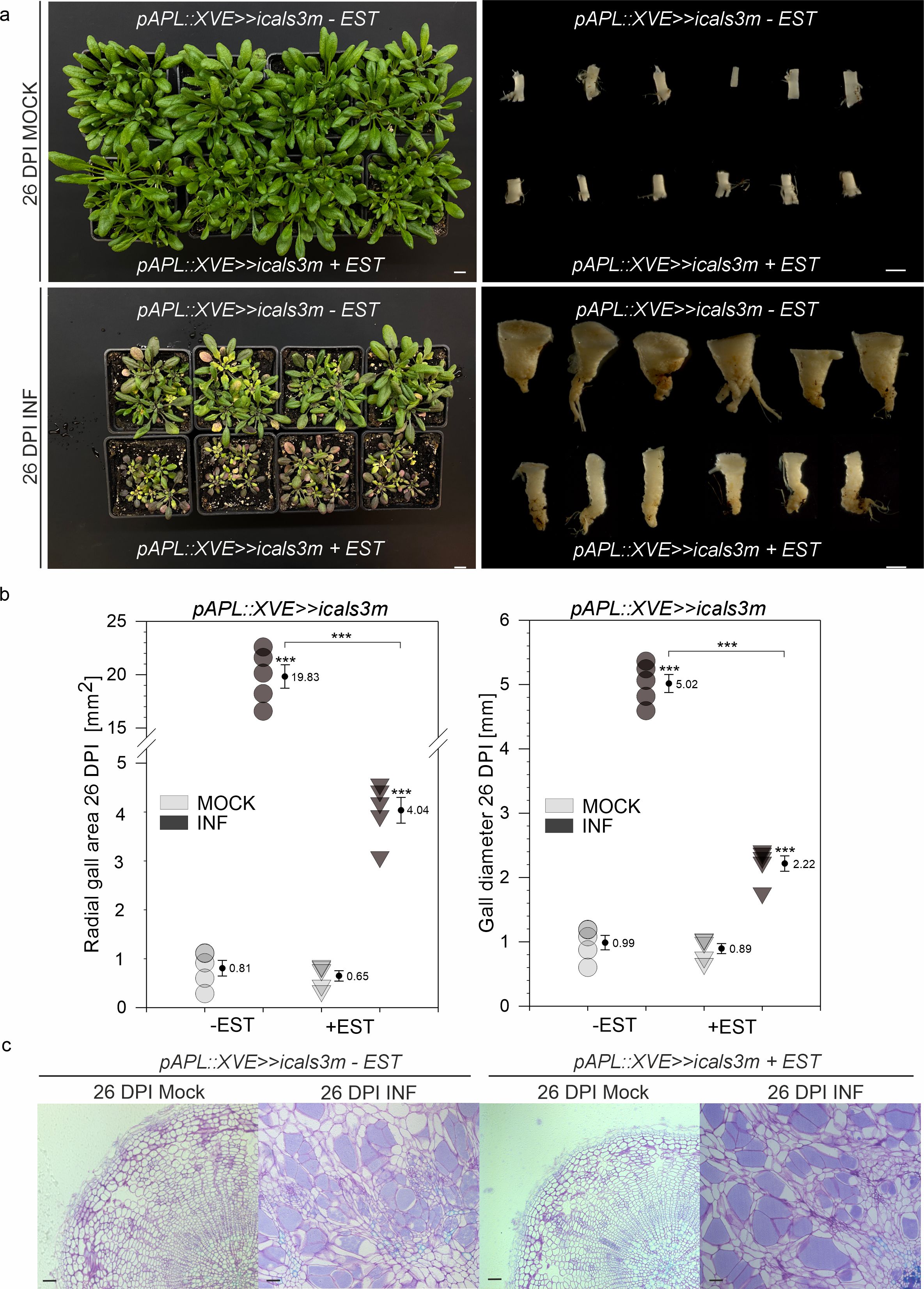

Supplement: Supplementary file 3 — Figure S3. Effects of phloem transport disruption on Plasmodiophora brassicae disease severity and gall development studied in estradiol inducible pAPL::XVE>>icals3m line. Panel (a) shows differences in the phenotype of rosettes and hypocotyls observed in pAPL::XVE>>icals3m line upon P. brassicae infection between plants depositing callose in phloem SE cells (+EST) and plants having uncompromised phloem transport (−EST). Panel (b) shows the comparison for hypocotyl diameter. Panel (c) shows clubroot disease severity comparison at 26 DPI. Radial sections were stained with TB solution. [file TPJ-121-0-s009.jpg]

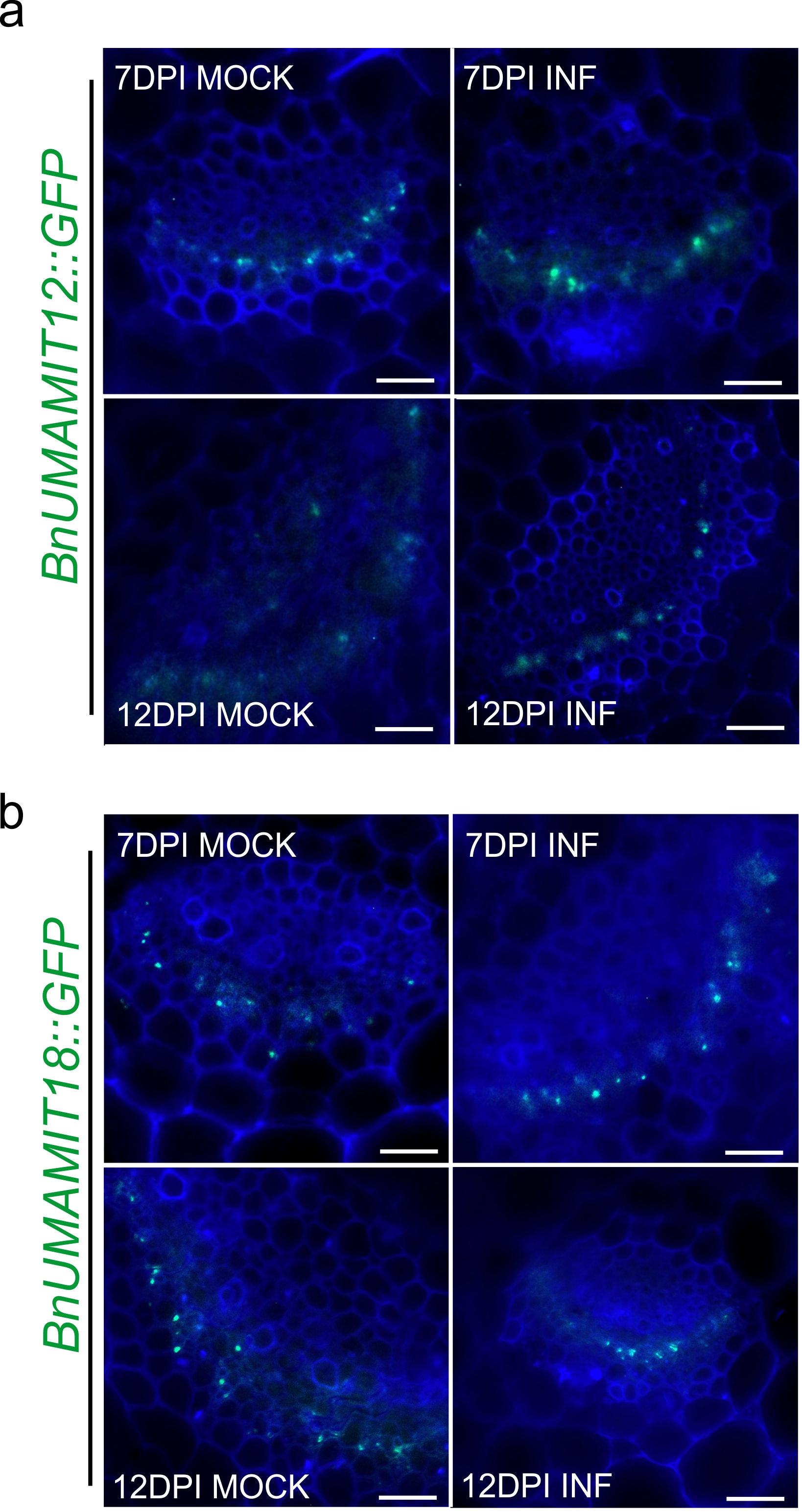

Supplement: Supplementary file 4 — Figure S4. Promoter activity for GFP fusions of UMAMIT 12 and 18 Brassica napus amino acid transporters orthologues A05p08480.1_BnaDAR and C08p07150.1_BnaDAR respectively at 7 and 12 DPI in transverse sections of petioles from mock‐treated and Plasmodiophora brassicae‐infected Arabidopsis plants. Sections were counterstained with Calcofluor White and GFP signals from reporter genes fused to chosen promoter were observed under an epifluorescent microscope. Scale bars represent 50 μm. [file TPJ-121-0-s005.jpg]

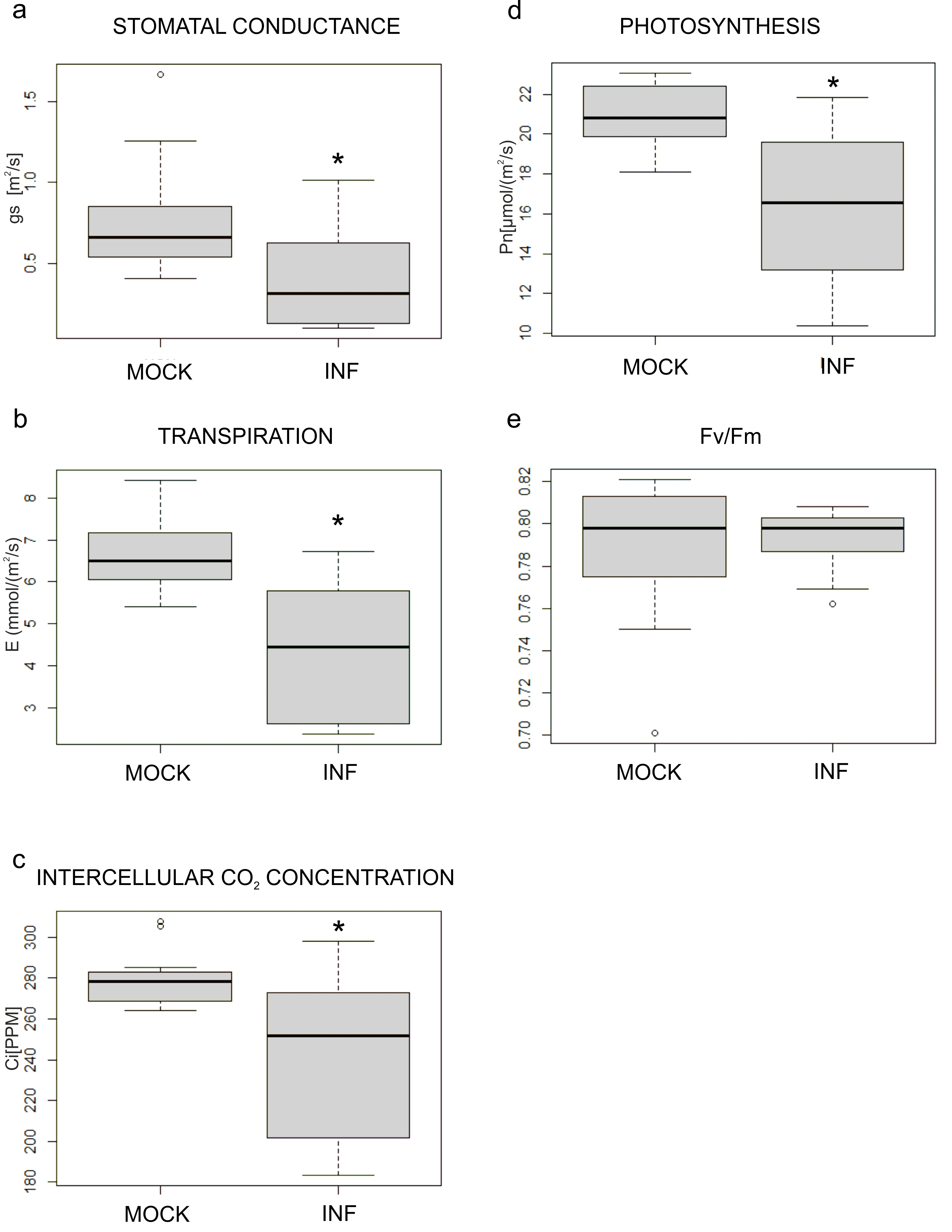

Supplement: Supplementary file 5 — Figure S5. Comparison of photosynthesis and gas exchange parameters in Plasmodiophora brassicae‐infected OSR plants versus appropriate mock‐inoculated control at 12 DPI. Panel (a) shows stomatal conductance (n = 10), (b) transpiration rate (n = 10), (c) intercellular CO2 concentration (n = 10), (d) photosynthetic efficiency (n = 10) and (e) maximum photochemical efficacy F v/F m (n = 20). Asterisks indicate statistically significant differences at P ≤ 0.05 that were calculated by unpaired t‐test for results that showed a normal distribution, or by Wilcoxon signed‐rank test for data that do not follow normal distribution. [Correction added on 6 December 2024, after first online publication: the legends of Figures S4 and S5 have been swapped in this version.] [file TPJ-121-0-s002.jpg]

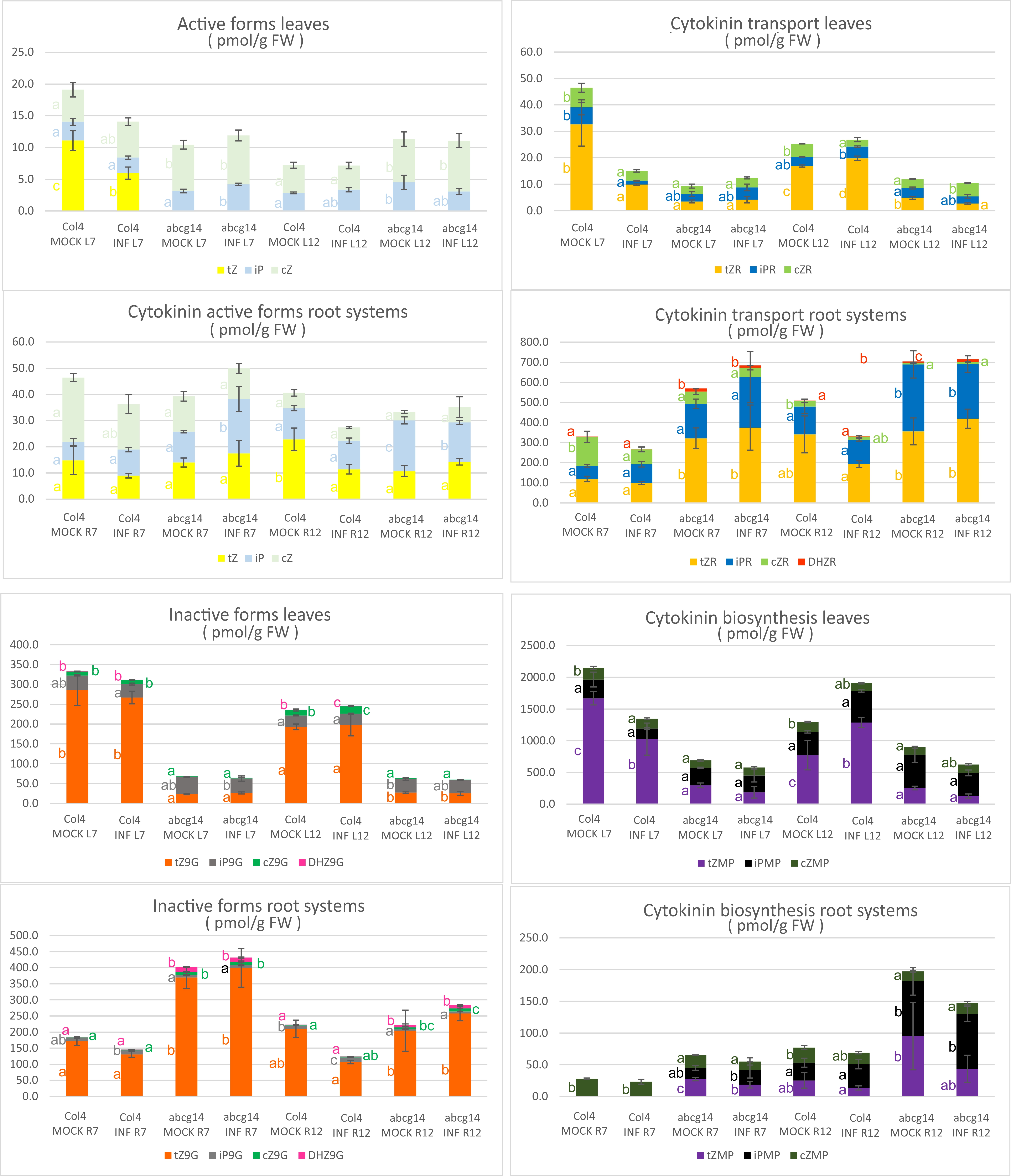

Supplement: Supplementary file 6 — Figure S6. Cytokinin changes triggered by Plasmodiophora brassicae in Col‐4 and abcg14 mutant. Detected components were divided into four groups based on their function in the plant—active forms (trans‐zeatin tZ, cis‐zeatin cZ and isopentenyladenine iP), inactive forms (trans zeatin glucoside tZ9G, cis zeatin glucoside cZ9G, isopentenyl adenine glucoside iP9G and di‐hydroxy zeatin glucoside DHZ9G), transport forms (trans‐zeatin riboside tZR, cis‐zeatin riboside cZR and isopentenyl riboside iPR) and primary components of cytokinin biosynthesis (trans‐zeatin monophosphate tZMP, cis‐zeatin monophosphate cZMP and isopentenyl monophosphate iPMP). Error bars represent SD values of 3 biological replicates (at 7 DPI time points each biological replicate included 60 plants for Col‐4 and 90 plants for abcg14. At 12 DPI time points 36 plants represent 1 biological repeat). Different letters indicate a significant difference between means for the particular cytokinin form according to one‐way ANOVA with Tukey's multiple comparison test. [file TPJ-121-0-s001.jpg]

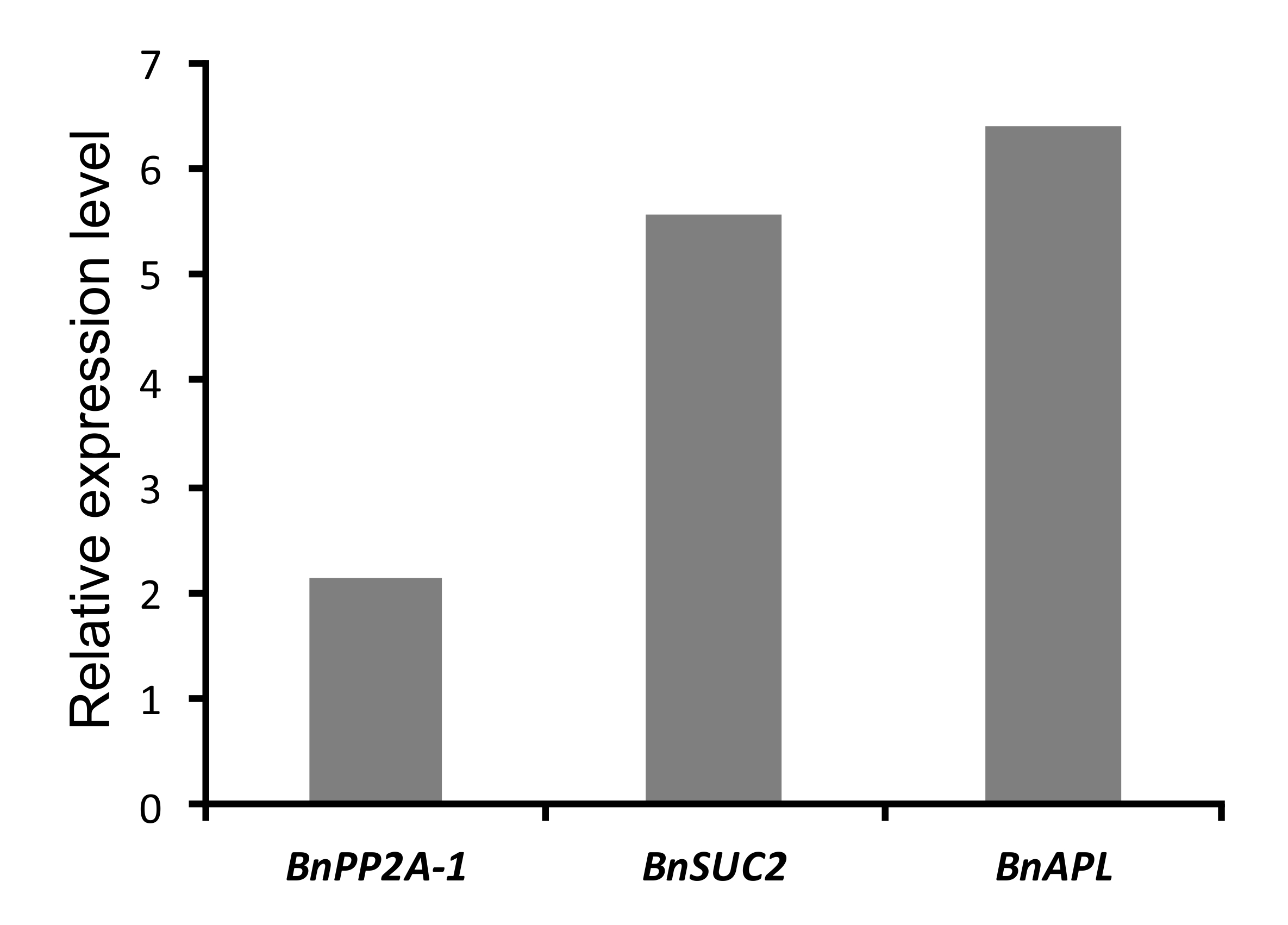

Supplement: Supplementary file 7 — Figure S7. Phloem‐specific gene enrichment test performed for dissected material. Relative expression levels of the BnPP2A‐1, BnSUC2 and BnAPL genes were higher in dissected phloem regions in comparison to the surrounding tissue material. [file TPJ-121-0-s006.jpg]
