# Supplementary material for: Laser dissection‐assisted phloem transcriptomics highlights the metabolic and physiological changes accompanying clubroot disease progression in oilseed rape
Source: Plant J. 2024 Nov 22;121(1):e17156. doi: 10.1111/tpj.17156 (PMC11703547; doi:10.1111/tpj.17156)
Supplement: Supplementary file 8 — Table S1. List of oligonucleotide primers used for the enrichment test of laser dissected material before cDNA libraries preparation and transcript sequencing. [file TPJ-121-0-s004.docx]

**Supplementary Table S1**

List of oligonucleotide primers used for the enrichment test of laser dissected material before cDNA libraries preparation and transcript sequencing

| Gene ID | Primer name | Primer sequence |
| --- | --- | --- |
| A09p17880.1_BnaDAR | PP2A-1-F | AGATCGAGCAGCTGATGGAGTGT |
|  | PP2A-1-R | CATAGAACTGGCCGTGGATATCG |
| *A09p42030.1*_BnaDAR | SUC2-F | TTGGGGCTTATGCTTAACGC |
|  | SUC2-R | TTTGCTCCACCCATTTTCCG |
| *A07p44500.1*_BnaDAR | APL-F | ATTCAGCGCAACGTAGCTTC |
|  | APL-R | AGCTGCAAATGTCTCTGCAC |
| C08p10490.1_BnaDAR | UBC9-F | TCCATCCGACAGCCCTTACTCT |
|  | UBC9-R | ACACTTTGGTCCTAAAAGCCACC |
| *A10p10830.1*_BnaDAR | UBC10-F | GAAGACATGTTTCATTGGCAGGC |
|  | UBC10-R | CTTAGGAGGTTTGAAAGGGTAATCA |
| C02p04090.1_BnaDAR | ACT7-F | CTATCCTCCGTCTCGATCTCGC |
|  | ACT7-R | CTTAGCCGTCTCCAGCTCTTGC |
| *A01p06100.1*_BnaDAR | ENTH-F | GTTTAGACCCGTTGCTGCTC |
|  | ENTH-R | TTGTCCATCTCAGCCATTTG |
