# Supplementary material for: Laser dissection‐assisted phloem transcriptomics highlights the metabolic and physiological changes accompanying clubroot disease progression in oilseed rape
Source: Plant J. 2024 Nov 22;121(1):e17156. doi: 10.1111/tpj.17156 (PMC11703547; doi:10.1111/tpj.17156)
Supplement: Supplementary file 9 — Table S2. List of oligonucleotide primers used for 5′ upstream sequences cloning and preparation of constructs in a GreenGate system. [file TPJ-121-0-s007.docx]

**Supplementary Table S 2**

List of oligonucleotide primers used for 5’upstream sequences cloning and preparation of constructs in a GreenGate system

| Gene ID | Primer name | Primer sequence (overhangs are labelled with colour) | Amplified sequence length |
| --- | --- | --- | --- |
| *C07p51310.1*_*BnaDAR* | proNRT1.8-F | AACAGGTCTCAACCTTTCGCAATCAAAGACAGCTA | 1452 bp |
|  | proNRT1.8-R | AACAGGTCTCATGTTCTATGTTGAAAAAGATTCCTTATCTTGA |  |
| *A05p08480.1*_*BnaDAR* | proUMAMIT12-F | AACAGGTCTCAACCTTCTTTGCAAATATCGTAGTAGC | 1494 bp |
|  | proUMAMIT12-R | AACAGGTCTCATGTTCTTAATAGCTTAAGATCAACAATCTCC |  |
| *C08p07150.1*_*BnaDAR* | proUMAMIT18-F | AACAGGTCTCAACCTTATTGGAAAATCCAAGAATTAATGTG | 1435 bp |
|  | proUMAMIT18-R | AACAGGTCTCATGTTCTTTGTTTGTTGATTAGTTTTAACACT |  |
| *A09p18320.1*_*BnaDAR* | proAAP1-F | AACAGGTCTCAACCTCTTAAACATTTTGTTTGGCAAGTA | 1496 bp |
|  | proAAP1-R | AACAGGTCTCATGTTAGTGAGAGAAGAGAGCAAAG |  |
